# Sulindac Compounds Facilitate the Cytotoxicity of β-Lapachone by Up-Regulation of NAD(P)H Quinone Oxidoreductase in Human Lung Cancer Cells

**DOI:** 10.1371/journal.pone.0088122

**Published:** 2014-02-05

**Authors:** Hsiu-Ni Kung, Tsai-Yun Weng, Yu-Lin Liu, Kuo-Shyan Lu, Yat-Pang Chau

**Affiliations:** 1 Department of Anatomy and Cell Biology, College of Medicine, National Taiwan University, Taipei, Taiwan; 2 Institute of Anatomy and Cell Biology, School of Medicine, National Yang-Ming University, Taipei, Taiwan; 3 Department of Medicine, Mackay Medical College, New Taipei City, Taiwan; Hungarian Academy of Sciences, Hungary

## Abstract

β-lapachone, a major component in an ethanol extract of *Tabebuia avellanedae* bark, is a promising potential therapeutic drug for various tumors, including lung cancer, the leading cause of cancer-related deaths worldwide. In the first part of this study, we found that apoptotic cell death induced in lung cancer cells by high concentrations of β-lapachone was mediated by increased activation of the pro-apoptotic factor JNK and decreased activation of the cell survival/proliferation factors PI3K, AKT, and ERK. In addition, β-lapachone toxicity was positively correlated with the expression and activity of NAD(P)H quinone oxidoreductase 1 (NQO1) in the tumor cells. In the second part, we found that the FDA-approved non-steroidal anti-inflammatory drug sulindac and its metabolites, sulindac sulfide and sulindac sulfone, increased NQO1 expression and activity in the lung adenocarcinoma cell lines CL1-1 and CL1-5, which have lower NQO1 levels and lower sensitivity to β-lapachone treatment than the A549 cell lines, and that inhibition of NQO1 by either dicoumarol treatment or NQO1 siRNA knockdown inhibited this sulindac-induced increase in β-lapachone cytotoxicity. In conclusion, sulindac and its metabolites synergistically increase the anticancer effects of β-lapachone primarily by increasing NQO1 activity and expression, and these two drugs may provide a novel combination therapy for lung cancers.

## Introduction

β-Lapachone, a natural o-naphthoquinone originally obtained from *lapacho* trees in South America, has promising anti-tumor activity on various tumor cells [1]-[6] and has been tested as an anti-tumor candidate drug in phase I/II/III clinical trials in combination with other chemotherapy drugs [1], [7]. Its anti-cancer activity is thought to be due to the two-electron reduction of β-lapachone catalyzed by NAD(P)H : quinone oxidoreductase (NQO1, DT-diaphorase), using NAD(P)H or NADH as electron source [1], [8], [9]. In the presence of NQO1, β-lapachone undergoes reduction to an unstable hydroquinone, which rapidly undergoes a two-step oxidation back to the parent compound, perpetuating a futile redox cycle and resulting in the generation of reactive oxygen species (ROS) including superoxides [8], [10]–[12]. These reactive species can oxidize thiol groups of the mitochondrial potential transition pore complex, leading to increased mitochondrial inner membrane permeability, reduced mitochondrial membrane depolarization, and release of cytochrome c, resulting in cell death [13], [14]. Because NQO1 is more highly expressed in various solid cancers than in normal tissues [15], β-lapachone can selectively kill these cancer cells. In addition, higher NQO1 expression or activity in cancer cells may make them more sensitive to β-lapachone. In order to increase the clinical efficacy of β-lapachone, many methods have been examined to increase NQO1 expression or activity in cancer cells [3], [5], [16]–[19].

Sulindac is a Food and Drug Administration (FDA)-approved non-steroidal anti-inflammatory drug (NSAID) for the treatment of osteoarthritis, ankylosing spondylitis, gout, or rheumatoid arthritis [20]–[23]. Its anti-inflammatory activity is due to its inhibition of the synthesis of prostaglandins [24], which cause inflammation and pain in the body. Sulindac has also been found to block cyclic guanosine monophosphate-phosphodiesterase, an enzyme that inhibits the normal apoptosis signaling pathway, and this inhibitory effect allows the apoptotic signaling pathway to proceed unopposed, resulting in apoptotic cell death and reducing the incidence of various tumors, including breast, esophageal, stomach, prostate, bladder, ovary, and lung cancers [25], [26]. In humans, sulindac is reduced to the active anti-inflammatory metabolite, sulindac sulfide undergoes a 2-step reoxidation to sulindac sulfone [27], [28]. All three compounds have been shown to have chemoprotective effects. In colon cancer, sulindac has been used to increase the anticancer effects of some reagents or stresses, including bortezomib [4], hydrogen peroxide [29], and oxidative stress [30]. Importantly, sulindac and its metabolites modulate the expression of multioxidative enzymes, including glutathione S-transferases and NQO1, the latter being the key regulator of β-lapachone-induced cell death in cancer cells [28], [31], [32], and sulindac might therefore have a synergistic anti-tumor effect with β-lapachone.

Lung cancer, the major cancer worldwide, is now the leading cause of cancer-related deaths [33]–[35]. According to a report of the Department of Health, Executive Yuan, ROC (Taiwan) published in 2010, the mortality rate for lung cancer is 20%, topping the list of all cancer-related deaths. The cost of health care for treatment of lung disease is increasing tremendously each year and threatens to overwhelm public health services [36]. In order to get a better target therapy, researchers have tried to identify key differences between lung cancer cells and normal lung cells, such as mutation or overexpression of genes, including *EGFR, ras*, and *VEGF* [37]–[39]. Unfortunately, current chemotherapies for lung cancer lack adequate specificity, efficacy, and treatment heterogeneity is also a big issue [40]. There is therefore an urgent need for new therapeutic drugs or new combinations of drugs to provide more efficient lung cancer therapy. Since NQO1 overexpression has been noted in both non-small cell lung cancer (NSCLC) cell line [41], [42], β-lapachone could be a potential therapeutic drug for lung cancers. However, some lung cancer cells show lower NQO1 expression or activity and might therefore be resistant to β-lapachone toxicity. In this study, we first investigated the relationship between β-lapachone toxicity and NQO1 levels in NSCLC cell lines, then determined the signaling pathway involved in the cell death caused by high concentrations of β-lapachone. We also used lower concentrations of β-lapachone to explore whether sulindac and its metabolites could facilitate the anticancer effect of β-lapachone by increasing NQO1 expression or activity in lung cancer cell lines with low NQO1 levels and checked the importance of NQO1 in this combination therapy. We found that the toxicity of β-lapachone was related to the level of NQO1 expression or activity in lung cancer cells and that high concentrations of β-lapachone killed cells by decreasing phosphorylation of PI3K, AKT, and ERK and activating JNK. In addition, the cytotoxicity of low concentrations of β-lapachone was increased by combination with sulindac and its metabolites, a process involving upregulation of expression or activity of NQO1.

## Materials and Methods

### Cell Culture

The human lung cancer cell lines CL1-1, CL1-5, and A549, were cultured in 5% CO_2_ at 37°C in RPMI 1640 medium containing 10% fetal calf serum, 100 Units/ml of penicillin, and 100 mg/ml of streptomycin (all from Gibco). The cell lines were gifts from Dr. PC Yang, National Taiwan University Hospital [43], in whose laboratory CL1-5 cells were selected from parental CL1-1 cells for greater metastatic potential using a transwell system.

### Cell Viability Assays

CL1-1, CL1-5, or A549 cells (1x10^4^) were seeded for 24 h at 37°C in a 96-well culture plate, then were subjected to starvation for 14 h in RPMI 1640 medium containing 2% fetal calf serum, 100 Units/ml of penicillin, and 100 mg/ml of streptomycin. Following 6 h pretreatment with medium or the indicated concentration of sulindac or its metabolites (all from Sigma), the cells were incubated for 12 h with or without the indicated concentration of β-lapachone in the continued presence of sulindac or its metabolites, and then cell viability was evaluated.

Two cell viability assays were used. In the crystal violet staining assay, the cells were fixed with 4% paraformaldehyde for 15 min, stained with 0.4% crystal violet for 15 min, and washed with H_2_O, then 50% acid alcohol was used to dissolve the bound crystal violet and the OD at 550 nm measured on an ELISA reader. In the MTT assay, 10 µl of MTT (0.5 mg/ml) (Sigma) was added to each well and the plates incubated at 37°C for 4 h, then the formazan product was dissolved in 100 µl of DMSO at 37°C for 30 min and the OD at 570 nm measured on a microplate reader.

### Acridine Orange (AO) Staining

Cells (5x10^4^) cultured on cover-slides in 24-well plates were incubated for 14 h in RPMI 1640 medium containing 2% fetal calf serum, preincubated with sulindac sulfide for 6h, and then treated with or without β-lapachone for 24 h, then were immediately fixed in 4% paraformaldehyde in phosphate-buffered saline (PBS) for 10 min at room temperature (RT), and stained for 10 min with 0.5 ml of AO (10 mg/ml in PBS) (Sigma). After several PBS washes, the cells were examined on an Olympus BH-2 inverted microscope equipped with a fluorescence attachment.

### Detection of Apoptosis and Measurement of Intracellular Calcium Levels

To detect apoptosis, cells (1x10^6^) were treated for 3, 6, or 9 h with 5 µM β-lapachone, then were washed with ice-cold PBS, trypsinized with 0.05% trypsin-0.02% EDTA, stained for 15 min at 37°C with Annexin V-FITC (10 µg/ml) (Strong Biotech Corporation, AVK050, Taipei, Taiwan), and analyzed by flow cytometry on a FACScan flow cytometer (Becton Dickinson).

To measure intracellular calcium levels, the cells were incubated for 10 min at 37°C with 2 mM Fluo-4/AM (Molecular Probes), washed with PBS, trypsinized, and analyzed by FACSan flow cytometry using the FL1H parameter.

### Western Blot Analyses

Treated cells were lysed with RIPA buffer containing 10 µg/ml of protease inhibitor (Sigma), and then the lysate was centrifuged at 10,000×g for 15 min at 4°C and the supernatant collected for immunoblotting. The protein concentration was measured by the Bradford assay, and samples containing 20 µg of protein were separated by 10 or 12% SDS–PAGE, and then transferred to Immobilon-P membranes for 2 h at 200 V (Millipore) in a Trans-Blot Electrophoretic Transfer cell. The membranes were blocked for 1 h at RT with 5% skim milk in PBS-0.2% Tween 20 (PBS-T), then incubated for 2 h at RT with antibodies against NQO1 (Cell Signaling), PI3 kinase or p-PI3 kinase (Millipore), AKT or p-AKT (Epitomics), ERK, p-ERK, JNK, or p-JNK (Cell Signalling),GAPDH (Genetex) or β-actin (Abcam) diluted 1∶1000 in 1% BSA. After washing for 30 min at RT with PBST, the membranes were incubated for 1 h at RT with horseradish peroxidase-conjugated secondary antibody (Perkin-Elmer, Boston, MA; 1∶5000 dilution in PBST), then bound antibody was detected using the ECL Western blotting reagent (Amersham), chemiluminescence being detected using a Fuji Medical X-ray film (Tokyo, Japan) and quantified by gel image analyses with Image Pro software. The intensity of the band of interest was divided by that for β-actin or GAPDH (loading controls) and this value normalized to that seen with no treatment.

### RNA Interference

The cells were transfected with non-targeting control siRNA (siNeg) or siRNA targeting NQO1 (siNQO1) (Applied Biosystems) using XtremeGene siRNA transfection reagent (Roche), then levels of the indicated transcripts and proteins were examined by realtime PCR (using the primers listed in Table S1), RT-PCR, and Western blotting, and the cells were then used in experiments.

### Reverse Transcription-PCR

Total RNA was extracted with Trizol (invitrogen) and reverse-transcribed to cDNA using a SuperScript II reverse transcription kit (Invitrogen), then PCR was performed using the following primers: NQO1 (F, TCCTCAGAGTGGCATTCTGC;R, TCTCCTCATCCTGTACCTCT) or GAPDH (F, CAACTACATGGTTTACATGTTC;R, GCCAGTGGACTCCACGAC).

### NQO1 Activity Assay

To measure endogenous NQO1 activity in cell extracts, cells were washed with PBS and sonicated in lysis buffer (25 mM Tris, pH 7.5, 1 mM EDTA, 0.1 mM DTT). The assay reaction mixture (final volume 200 µl) contained 25 mM Tris pH 7.5, 0.01% Tween 20, 0.7 mg/ml of BSA, 40 µM 2,6-dichloroindophenol (DCPIP; Sigma), 5 µM FAD, 200 µM NADH, 50 µg of cell extract, and either 10 µM dicoumarol or medium. The decrease in DCPIP absorbance at 600 nm in the absence or presence of dicoumarol was measured at 5 sec intervals for 60 sec and the activity expressed as relative activity, with the control activity given a value of 1.

### Statistical Analysis

All quantitative data are presented as the mean ± SEM for at least three separate experiments. Differences between groups were examined using one-way ANOVA with Scheffe’s test, with p<0.05 (* or #), p<0.005 (**), or p<0.001 (***) being considered statistically significant.

## Results

### NQO1 Expression and Activity in Lung Cancer Cells Correlate with β-lapachone Toxicity

**Figure 1:**
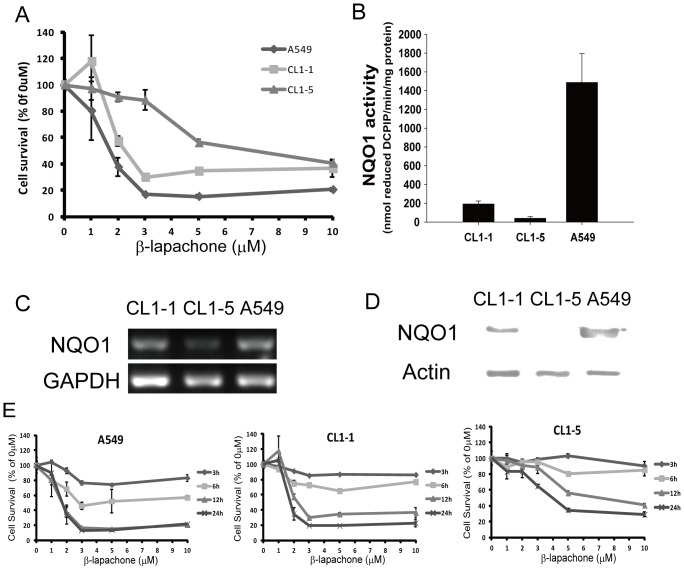
β-lapachone-induced cell death is associated with NQO1 expression levels. (A) Percentage survival of the lung cancer cell lines CL1-1, CL1-5, and A549. Cells were treated with 0–10 µM β-lapachone for 12 h, then cell viability was determined by crystal violet staining assay and expressed as a percentage of the value for cultures with no β-lapachone. (B–D) NQO1 activity levels (B), NQO1 RNA expression levels (C), and NQO1 protein expression levels (D) in the three lung cancer cell lines grown under normal culture conditions. (E) Percentage survival of A549 cells (left panel), CL1-1 cells (center panel), and CL1-5 cells (right panel) incubated with the indicated concentration of β-lapachone for 3, 6, 12, or 24 h examined by crystal violet staining and expressed as percentage survival compared to the untreated cells. The results are the mean ± SD for 3 independent experiments, each in triplicate.

To compare the cytotoxicity of β-lapachone for various lung cancer cells, three cell lines, CL1-1, CL1-5, and A549, were incubated for 12 h with β-lapachone (0 to 10 µM), then cell survival was measured by crystal violet staining. As shown in Figure 1A, using 1-5 µM β-lapachone, the A549 cells showed a significantly lower percentage survival than the CL1-1 and CL1-5 cells, but there was no significant difference between the different cells using 10 µM β-lapachone. Since NQO1 activity has been positively correlated with β-lapachone cytotoxicity in breast cancer cell lines [8], [9], [44], we examined whether the sensitivity of the different lung cancer cell lines to β-lapachone toxicity was associated with intracellular NQO1 expression. Figures 1B-D showed NQO1 activity (Fig. 1B), NQO1 RNA levels (Fig. 1C), and NQO1 protein levels (Fig. 1D) in CL1-1, CL1-5, and A549 cells and showed that, under normal culture conditions, all three values were highest in A549 cells and lowest in CL1-5 cells. We then compared the sensitivity of A549, CL1-1, and CL1-5 cells to treatment with 0–10 µM β-lapachone for 3, 6, 12, or 24 h using crystal violet staining and found that the percentage survival of CL1-5 cells was higher than that of CL1-1 cells at all 4 time points, and that A549 cells showed the lowest percentage survival (Figure 1E). These results corresponded well with the intracellular NQO1 levels in the three cell lines, as the sensitivity to β-lapachone was higher in cells with higher NQO1 levels. These data showed that NQO1 level or activity plays a key role in β-lapachone cytotoxicity for lung cancer cell lines.

In subsequent experiments, since β-lapachone alone was very effective at killing A549 cells and we wished to examine whether sulindac or its metabolites had a synergistic effect with β-lapachone, we concentrated on CL1-1 and CL1-5 cells. In addition, since the largest difference in survival of CL1-1 and CL1-5 cells was seen at β-lapachone concentrations of 2 to 5 µM, we used 5 µM β-lapachone to study the effect of β-lapachone alone and 2 µM β-lapachone to study synergistic effects of sulindac and β-lapachone.

### Identification of the Apoptotic Signaling Pathway Triggered by β-lapachone

**Figure 2:**
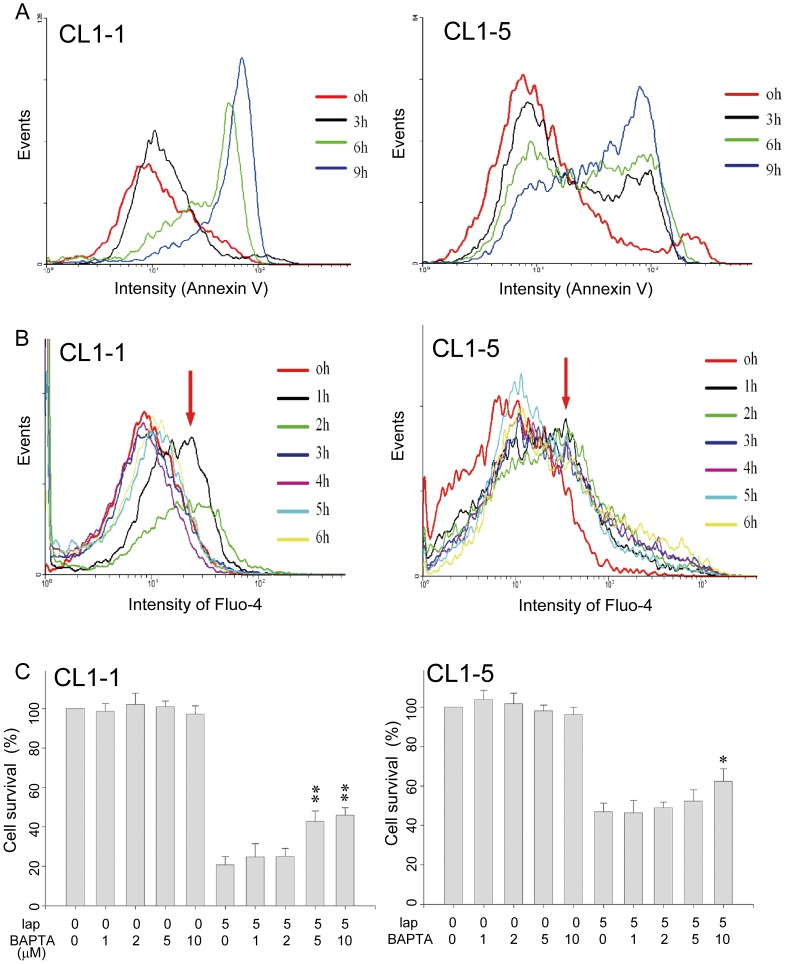
The β-lapachone-induced apoptosis of CL1-1 and CL1-5 cells is partly due to an intracellular calcium increase. (A) CL1-1 cells (left panel) or CL1-5 cells (right panel) were incubated with 5 µM β-lapachone for 0, 3, 6, or 9 h, then were examined for apoptosis using Annexin V. (B) CL1-1 cells (left panel) or CL1-5 cells (right panel) were incubated with 5 µM β-lapachone for the indicated time, then intracellular calcium levels were measured using Fluo-4 staining and flow cytometry. The intensity of Fluo-4 staining was increased by β-lapachone treatment, especially at 1 h (arrows). (C) CL1-1 cells (left panel) or CL1-5 cells (right panel) were left untreated or were incubated for 24 h with the indicated concentration of BAPTA-AM, an intracellular calcium chelator, and/or 5 µM β-lapachone, then cell survival was measured by the MTT assay and expressed as percentage survival compared to the untreated cells. * p<0.05, ** p<0.01 as compared to β-lapachone alone.

To investigate the underlying mechanism involved in β-lapachone toxicity, 5 µM β-lapachone was used to explore the apoptotic signaling pathway activated by β-lapachone in CL1-1 and CL1-5 cells. Using Annexin V staining, cell death in β-lapachone-treated CL1-1 and CL1-5 was demonstrated to occur by apoptosis (Figure 2A). Cell cycle analysis also showed that the sub G0/G1 ratio (apoptotic cells) increased in a time-dependent manner (Figure S1A). In studies measuring intracellular calcium levels, an increase was seen after 1 or 2 h of β-lapachone treatment in both CL1-1 and CL1-5 cells (Figure 2B, arrow), as seen during activation of the apoptotic pathway by β-lapachone [6], [45]. The percentage cell survival, measured using the MTT assay, was only partially restored by addition of 0-10 µM BAPTA (Molecular Probes), an intracellular calcium chelator, during incubation for 24 h with 5 µM β-lapachone (Figure 2C), showing that increased intracellular calcium levels was not the only factor involved in the β-lapachone-induced cell death of these cells. Although calpain and caspase 3, components of the apoptotic signaling pathway, were activated by treatment with 5 µM β-lapachone for 0–9 h (Figure S1B), as shown in Supplementary Figure 2, caspases and calpain were not involved in the lung cancer cell death induced by β-lapachone, as 1 h pretreatment with the pan caspase inhibitor zVAD or the calpain inhibitor ALLM or ALLN (all from Sigma) did not inhibit the effect (Figure S2). In both cell lines, the mitochondrial membrane potential (MMP) was decreased by treatment for 3, 6, or 9 h with β-lapachone (Figure S1C), but intracellular H_2_O_2_ levels were not changed by β-lapachone treatment for 3 or 6 h (Figure S1D). These results shown that β-lapachone causes apoptosis of both CL1-1 and CL1-5 cells by decreasing the MMP.

**Figure 3:**
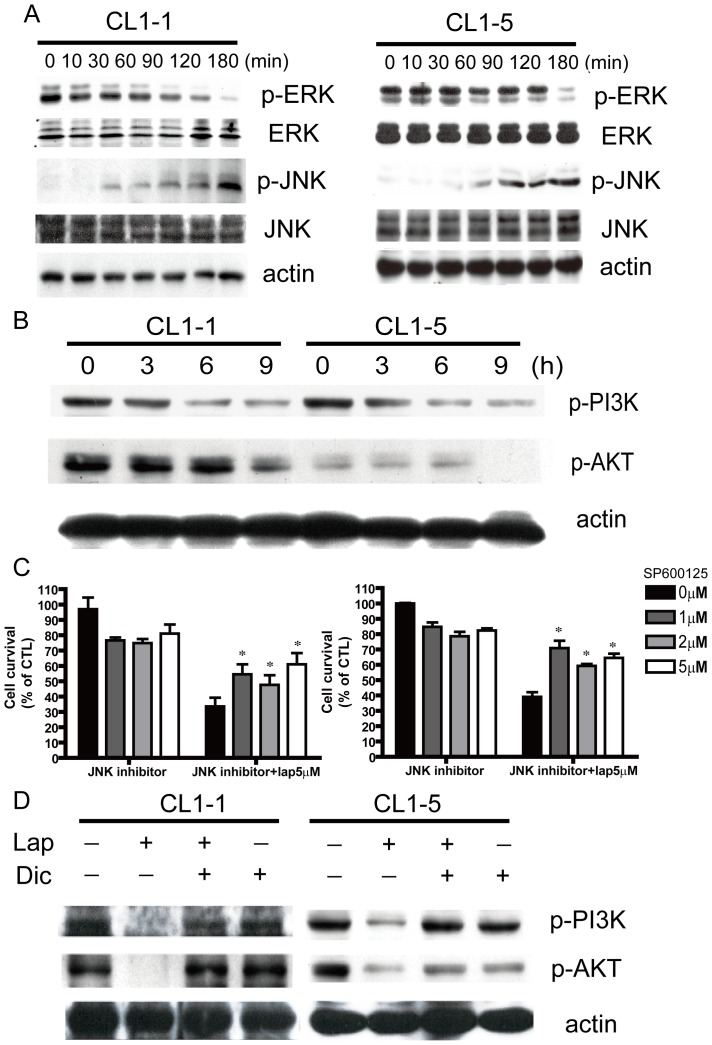
Signaling pathway components involved in β-lapachone-induced apoptosis. (A) CL1-1 cells (left) or CL1-5 cells (right) were incubated with 5 µM β-lapachone for the indicated time, then levels of p-ERK, ERK, p-JNK, and JNK were measured by Western blotting. (B) CL1-1 cells (left) or CL1-5 cells (right) were incubated with 5 µM β-lapachone for 0, 3, 6, or 9 h, then levels of p-PI3K and p-AKT were examined by Western blotting. (C) CL1-1 cells (left) or CL1-5 cells (right) were pretreated with the indicated concentrations of the JNK inhibitor sp600125 for 6 h, and then treated with or without 5 µM β-lapachone for 24 h.Cell survival was measured by the MTT assay and expressed as percentage survival compared to the untreated cells.* p<0.05 (D) CL1-1 cells (left) or CL1-5 cells (right) were left untreated or were preincubated for 1 h with 10 µM dicoumarol, then medium or 5 µM β-lapachone was added and the cells incubated for 9 h and levels of p-PI3K and p-AKT were measured by Western blotting.

To determine the signaling pathways activated in β-lapachone-induced lung cancer cell death, levels of the phosphorylated forms of PI3K, AKT, and the MAPKs ERK and JNK in CL1-1 and CL1-5 cells were examined. β-lapachone treatment for 10–180 min increased JNK phosphorylation, but decreased phosphorylation of ERK (Figure 3A) and of PI3K and AKT (Figure 3B). In addition, at concentrations of 1, 2, or 5 µM, the JNK inhibitor SP600125 partially rescued cells from toxicity induced by 24 h incubation with β-lapachone (Figure 3C), showing that JNK plays an important role in lung cancer cell death induced by β-lapachone.

**Figure 4:**
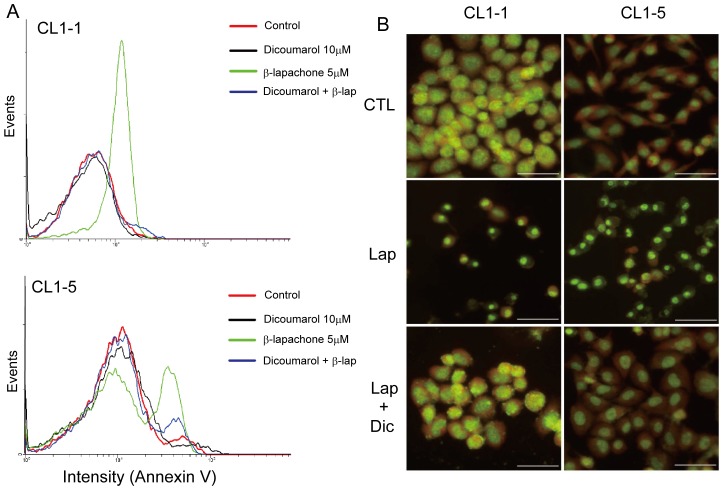
Dicoumarol, an NQO1 inhibitor, blocks the apoptotic effects of β-lapachone. (A) CL1-1 cells (top) or CL1-5 cells (bottom) were left untreated or were incubated for 6 h with 5 µM β-lapachone and/or 10 µM dicoumarol, then stained with Annexin V-FITC and the Annexin V fluorescence measured by flow cytometry. (B) Morphological changes after drug treatment. CL1-1 or CL1-5 cells were left untreated (CTL) or were incubated for 24 h with 5 µM β-lapachone with or without 10 µM dicoumarol, then stained with acridine orange to observe the morphology of the cell nucleus. The scale bar represents 50 µm.

In order to determine whether NQO1 was a key regulator in β-lapachone-mediated lung cancer cell death, cells were incubated for 6 h with 10 µM dicoumarol, a specific NQO1 inhibitor, and this resulted in about a 67% and 77% reduction in NQO1 activity in CL1-1 and CL1-5 cells, respectively (Figure S3A). Dicoumarol treatment significantly inhibited the decrease in phosphorylation of p-PI3K and p-AKT caused by 9 h of β-lapachone treatment (Figure 3D), blocked the increase in intracellular calcium levels induced by 1 h of β-lapachone treatment (Figure S3B), and markedly inhibited the apoptotic cell death caused by 6 h incubation with β-lapachone, as shown by Annexin V staining (Figure 4A) and acridine orange (AO) staining (Figure 4B).

### Sulindac and its Metabolites Increase the Cytotoxic Effect of β-lapachone through Activation of NQO1

The NSAID sulindac and its metabolites, sulindac sulfide (the reduced form) and sulindac sulfone (the oxidized form), are known to modulate the expression of some multioxidative enzymes, including NQO1 [31], [32]. Since NQO1 levels and activity were negatively associated with the cytotoxicity of β-lapachone, we next investigated whether sulindac and its metabolites could increase the cytotoxicity of β-lapachone for cells with low NQO1 expression and lower β-lapachone sensitivity, such as CL1-1 and CL1-5 cells.

**Figure 5:**
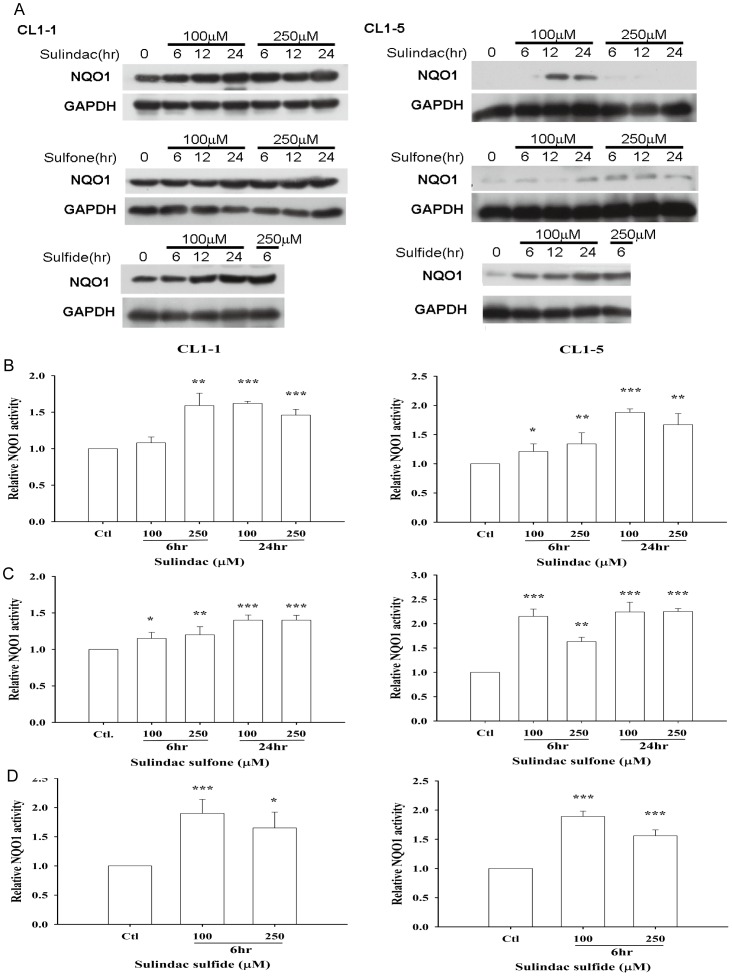
Sulindac and its metabolites increase NQO1 expression and activity. (A) CL1-1 cells (left) or CL1-5 cells (right) were left untreated or were incubated with 100 or 250 µM sulindac, sulindac sulfone, or sulindac sulfide for 6, 12, or 24 h, then protein levels were measured by Western blotting. (B–D) CL1-1 cells (left) or CL1-5 cells (right) were left untreated (Ctl) or were incubated with the indicated concentration of sulindac (B), sulindac sulfone (C), or sulindac sulfide (D) for the indicated time, then NQO1 activity was measured. * : p<0.05, **: p<0.01, ***: p<0.001 compared to the control.

**Figure 6:**
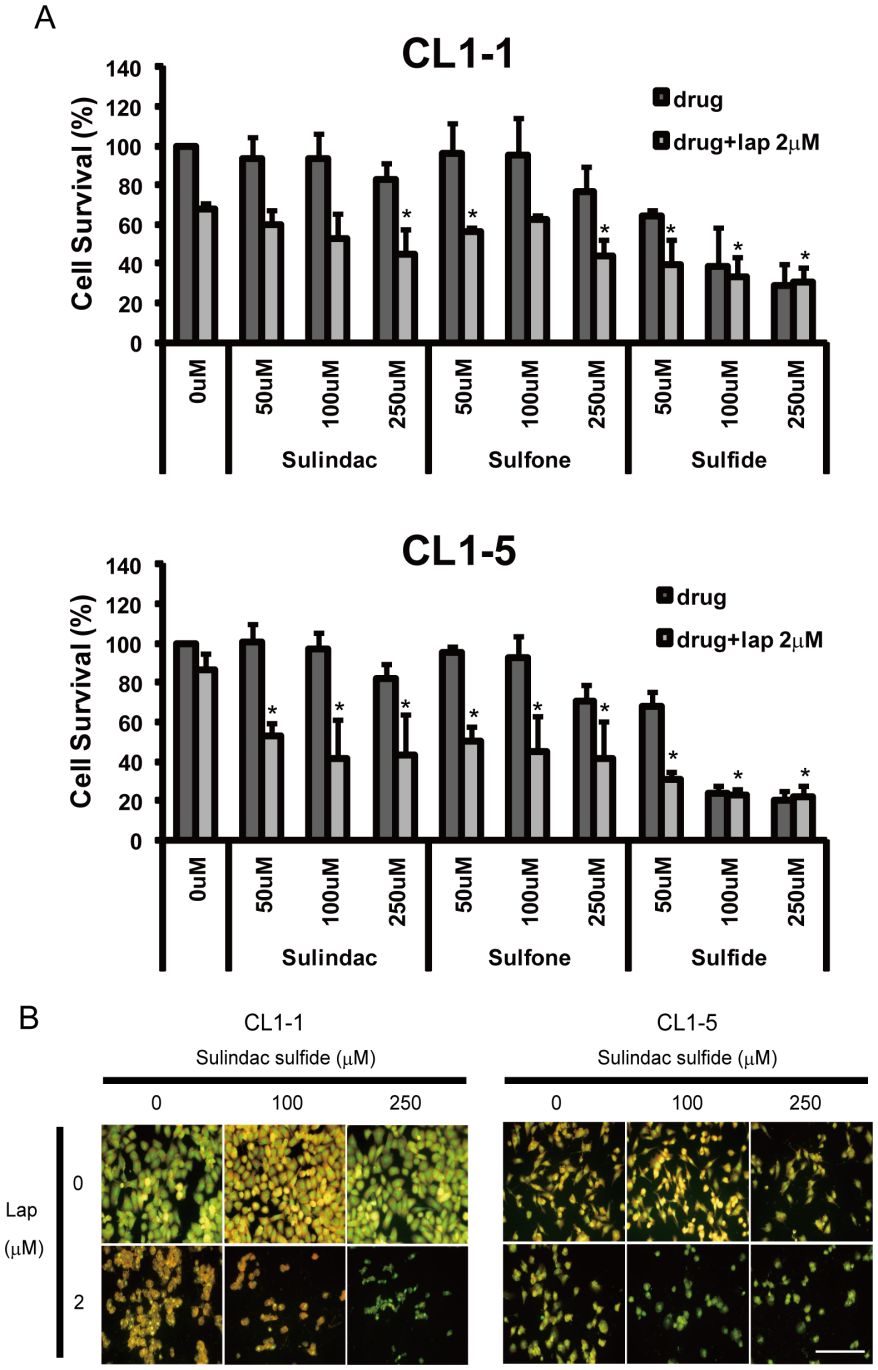
The cytotoxicity of β-lapachone for CL1-1 and CL1-5 cells is enhanced by sulindac and its metabolites. (A) CL1-1 cells (left) or CL1-5 cells (right) were left untreated or were pretreated for 6 h with the indicated concentration of sulindac, sulindac sulfone, and sulindac sulfide, then 2 µM β-lapachone was added for 12 h, then cell survival was measured using crystal violet staining and expressed as percentage survival compared to the untreated cells. * : p<0.05 compared to β-lapachone alone. (B) Two sets of each cell type were left untreated or were incubated for 6 h with 100 or 250 µM sulindac sulfide, then 2 µM B-lapachone was added to one set and incubation continued for 12 h, then the morphology was examined by acridine orange staining. The scale bar represents 100 µm.

To determine whether sulindac and its metabolites could modulate NQO1 expression in lung cancer cell lines, they were used at concentrations of 100 and 250 µM to treat CL1-1 and CL1-5 cells for 6, 12, or 24 h. As shown in Figure 5A, in CL1-1 cells, both concentrations of sulindac or metabolite upregulated NQO1 protein levels at all 3 time points, while, in CL1-5 cells, the results were more complex, an increase being seen after incubation for 12 or 24 h with 100 µM, but not 250 µM, sulindac, at all three time points with both concentrations of sulindac sulfone or 100 µM sulindac sulfide, and with 250 µM sulindac sulfide for 6 h (12 and 24 h not tested) (Figure 5A). NQO1 enzyme activity was also increased by all three chemicals (Figure 5B). As shown in Figure S4, at 100 and 250 µM, the three drugs had no significant effect on the percentage survival of CL1-1 and CL1-5 cells after incubation with sulindac or sulindac sulfone for 54 h or with sulindac sulfide for 12 h. In order to examine the synergistic effect of sulindac or its metabolites and β-lapachone in lung cancer cells, CL1-1 and CL1-5 cells were incubated with 0, 50, 100, or 250 µM sulindac or the metabolites for 6 h, then with 2 µM β-lapachone in the continued presence of sulindac or metabolite for 12 h, and the percentage survival was measured using crystal violet staining. Compared to cells treated with β-lapachone alone, the survival of both cell lines was decreased by 10-40% when cotreated with β-lapachone plus sulindac, 20-40% with β-lapachone plus sulindac sulfone, and 30-60% with β-lapachone plus sulindac sulfide (Figure 6A). The cotreatment-induced decrease was greater with CL1-5 cells than with CL1-1 cells, i.e. it was greater with the cells expressing lower NQO1 levels (Figure 6A); in addition, no additional effect of combined treatment compared to β-lapachone alone was seen with A549 cells (Figure S5) which express the highest NQC1 levels. These data show that sulindac can increase the sensitivity of cells with low NQO1 levels to β-lapachone cytotoxicity. Using AO staining and fluorescence microscopy, 6 h pretreatment with 100 or 250 µM sulindac sulfide, followed by addition of 2 µM β-lapachone for 12 h resulted in a decrease in CL1-1 and CL1-5 cell density compared to β-lapachone alone (Figure 6B), and similar results were obtained with the combination of β-lapachone and either sulindac or sulindac sulfone (data not shown).

### NQO1 Plays a Key Role in the Sulindac-induced Increase in β-lapachone Cytotoxicity for Lung Cancer Cells

Although NQO1 expression and activity were increased by sulindac and its metabolites, whether NQO1 was a major contributor to the sulindac-induced increase in β-lapachone cytotoxicity still required investigation. Two methods were used to inhibit the enzyme activity or protein expression of NQO1, an NQO1 inhibitor and NQO1 siRNA knockdown.

**Figure 7:**
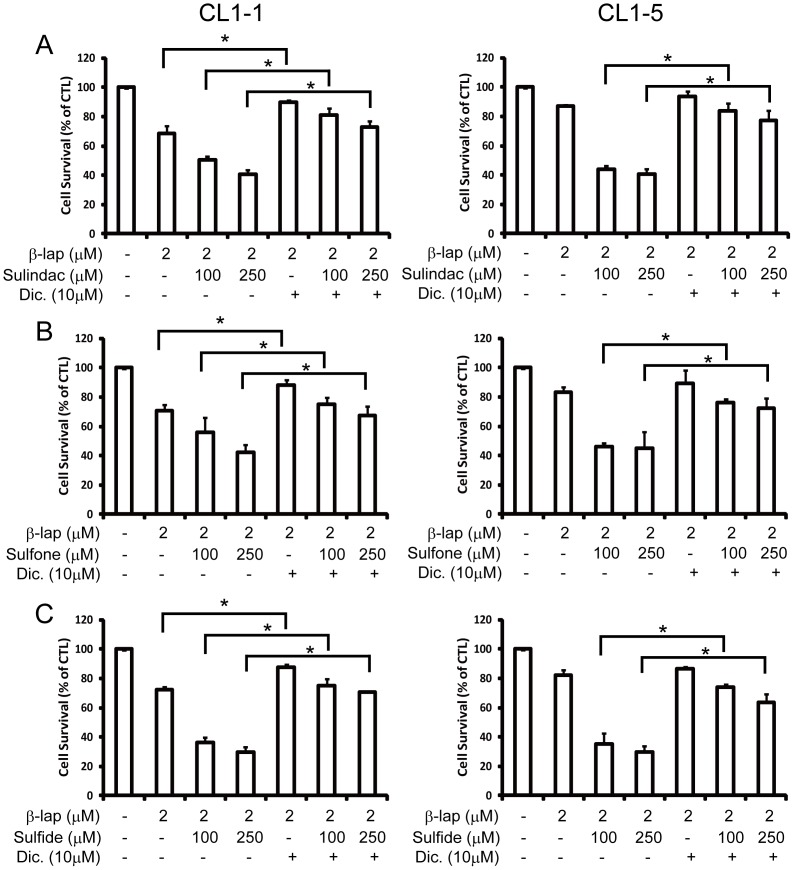
The increase in β-lapachone-induced cell death caused by sulindac and its metabolites is blocked by the NQO1 inhibitor, dicoumarol. CL1-1 cells (left) or CL1-5 cells (right) were left untreated or were pretreated for 6 h with 100 or 250 µM sulindac (A), sulindac sulfone (B), or sulindac sulfide (C) with or without 10 µM dicoumarol, then were incubated for a further 12 h with or without addition of 2 µM β-lapachone, then cell survival was measured by crystal violet staining and expressed as percentage survival compared to the untreated cells. * : p<0.05.

Dicoumarol has been previously used to specifically inhibit the expression and activity of NQO1 [44]. As shown in Figure 7, pretreatment of cells with 100 or 250 µM sulindac (Figure 7A), sulindac sulfone (Figure 7B), or sulindac sulfide (Figure 7C), followed by addition of 2 µM β-lapachone for 12 h increased the cytotoxicity of β-lapachone for both CL1-1 and CL1-5 cells and these effects were significantly reduced by addition of 10 µM dicoumarol.

**Figure 8:**
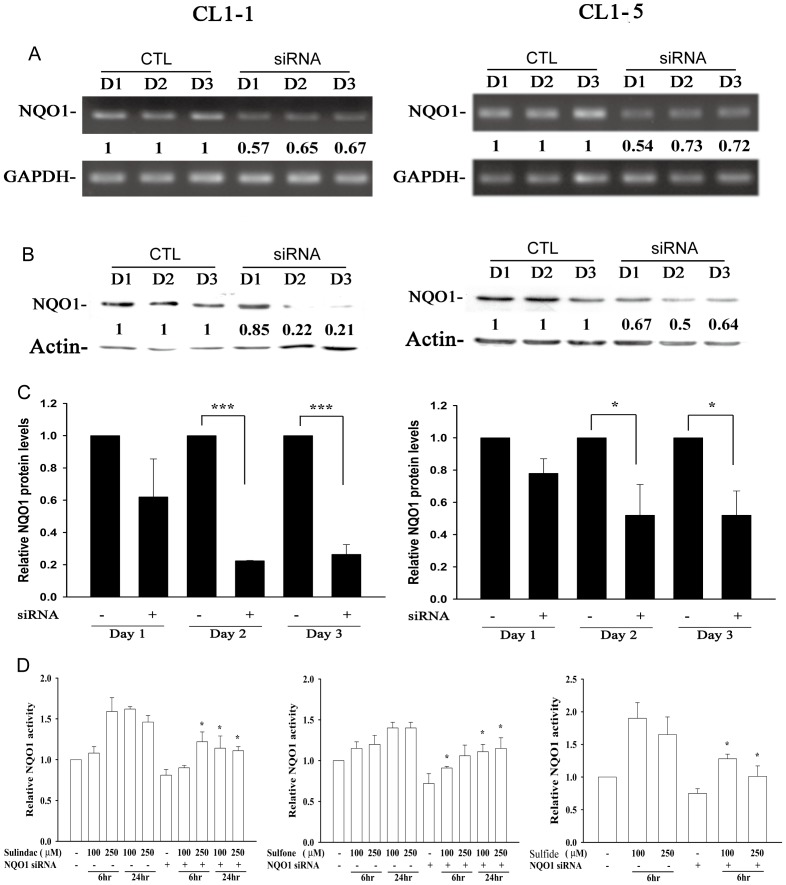
The knockdown effects of NQO1 siRNA on NQO1 RNA, protein, and activity. (A–C) CL1-1 cells (left) or CL1-5 cells (right) were transfected for 1 to 3 days with control siRNA (CTL) or siRNA targeting NQO1, then RNA expression was measured by PCR (A) and protein expression by Western blotting (B and C). (D) CL1-1 cells transfected for 2 days with control siRNA or NQO1 siRNA were incubated alone or with 100 or 250 µM sulindac, sulindac sulfide, or sulindac sulfone for 6 or 24 h, then NQO1 activity was measured. * : p<0.05, ***: p<0.001 compared to the identically treated cells transfected with control siRNA.

**Figure 9:**
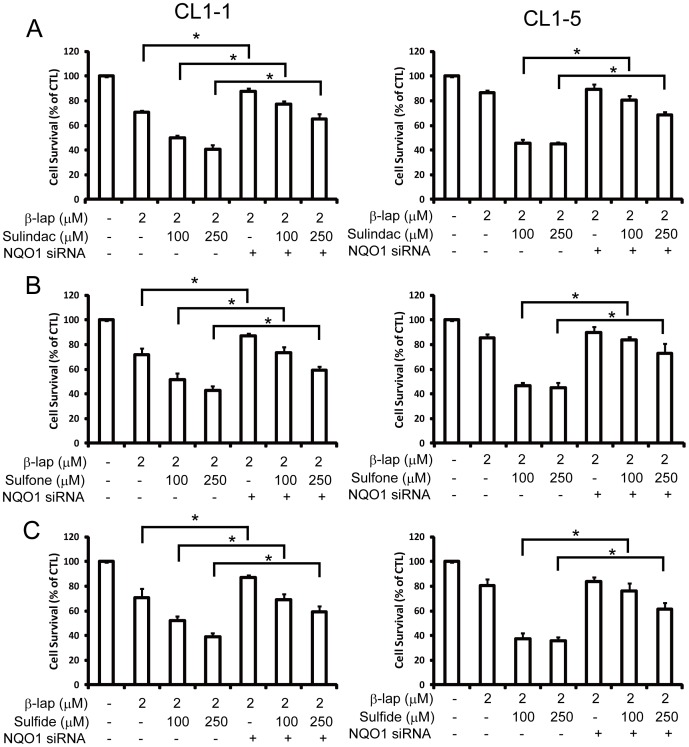
NQO1 siRNA transfection significantly inhibits the effect of sulindac and its metabolites on β-lapachone-induced cell death. CL1-1 cells (left) or CL1-5 cells (right) were transfected with control siRNA (−) or NQO1 siRNA (+) for 24 h, then were left untreated or were incubated for 6 h with 100 or 250 µM sulindac (A), sulindac sulfone (B), or sulindac sulfide (C), then 2 µM β-lapachone or medium was added and the cells incubated for 12 h, when cell survival was measured using crystal violet staining and expressed as percentage survival compared to the untreated cells. * : p<0.05 for the indicated comparison.

Using siRNA knockdown of NQO1, at days 1 to 3 after NQO1 siRNA transfection of CL1-1 and CL1-5 cells, no change in cell growth or cell morphology was noted (Figure S6). Efficiency of knockdown in CL1-1 and CL1-5 cells was demonstrated for RNA expression by RT-PCR (Figure 8A) and realtime-PCR (Figure S7) and for protein expression by western blotting (Figure 8B and C), showing that NQO1 siRNA significantly downregulated NQO1 expression. As shown in Figure 8D, NQO1 siRNA transfection significantly inhibited the increase in NQO1 enzyme activity induced in CL1-1 cells by incubation for 6 or 24 h with 100 or 250 µM sulindac (left panel), sulindac sulfone (center panel), or sulindac sulfide (right panel). When cells transfected for 24 h with siNQO1 or control siRNA were pretreated for 6 h with sulindac or its metabolites, then cotreated for 12 h with drug plus 2 µM β-lapachone, the percentage cell survival results showed results that transfection with NQO1 siRNA caused a significant decrease in the cytotoxicity of combinations of β-lapachone with sulindac (Figure 9A), sulindac sulfone (Figure 9B), or sulindac sulfide (Figure 9C). These results showed that NQO1 plays an important role in the increase in β-lapachone-induced cell death caused by sulindac or its metabolites.

## Discussion

### β-lapachone Triggers Lung Cancer Cells to Undergo Apoptosis through an Increase in Intracellular Calcium Levels, Increased JNK Activation, Decreased Activation of PI3K, ERK, and AKT, and a Decrease in the MMP

Cell proliferation and cell death are under complex and precise control. Normally, the proteins involved in cell proliferation, survival, or cell death are in a remarkable balance, and unbalanced survival and apoptotic signals may lead to cell death. In most cells, proliferation is mainly regulated through PI3K, AKT, and ERK [46], and cell death is regulated through another pathway involving JNK and p38 [47], [48]. JNK has recently been reported to be an important mediator in the β-lapachone-induced cell death of breast and prostate cancer cells [45], [49]. β-lapachone also triggers cell death of many cancer cells by increasing calcium signaling [6], [50]. Calcium, the key messenger molecule in cells, plays an important role in many signaling pathways and an imbalance in intracellular calcium levels causes abnormal cell function and leads to cell death. Treatment of cells with the intracellular calcium chelator BAPTA partially inhibited β-lapachone-induced cell death, showing that calcium was involved (Figure 2). Activation of the cell death signal JNK (Figure 3A) and inhibition of the cell survival signals, p-PI3K, p-AKT, and p-ERK (Figure 3A and B) were also observed in β-lapachone-mediated lung cancer cell death, showing that the MAP kinase signaling pathway is involved in the anticancer effect of β-lapachone. Although ROS could have caused the cell death induced by β-lapachone, there was no change in intracellular H_2_O_2_ levels following β-lapachone treatment (Figure S1D). However, the MMP was dramatically decreased following β-lapachone treatment (Figure S3C), suggesting that other ROS species might be involved in the β-lapachone-induced cell death process.

### NQO1 is a Key Factor in the β-lapachone-induced Lung Cancer Cell Death

High NQO1 activity and expression are seen in many human tumors, including carcinoma of the liver [51], [52], colon [53], breast [52], [54], brain [55], and lung [52], and NQO1 has been shown to be an important factor in β-lapachone-induced cell death in many kinds of cancer cells [9], [44], including breast cancer [2], glioma [56], and prostate cancer [44]. In this study, we demonstrated that the cytotoxicity of β-lapachone for three different lung cancer cell lines was positively correlated with their NQO1 expression and enzyme activity (Figure 1). Inhibition of NQO1 activity using the NQO1 inhibitor dicoumarol (Figure S3) blocked the β-lapachone-induced increase in intracellular calcium levels (Figure S3), increase in p-JNK levels (Figure 3A), and decrease in p-ERK, p-PI3K, and p-AKT levels (Figure 3A and B). These results indicated that the balance between survival and death signals in lung cancer cells was disrupted by the decrease in p-PI3K, p-AKT, and p-ERK levels and the increase in p-JNK levels caused by β-lapachone treatment, and that NQO1 expression and activity were involved in the activation of all these apoptotic signals.

### Anti-inflammatory Drugs Increase NQO1 Levels and Enzyme Activity in Lung Cancer Cells

Many drugs or treatments, such as cisplatin [5], heat shock [19], or radiation [57], can increase NQO1 expression or activity and facilitate the cytotoxicity of β-lapachone for various cancer cells. However, such drugs or treatments are usually harmful to normal cells as well as cancer cells, so there is a need for drugs or treatments that can facilitate the anti-cancer effect of β-lapachone, while being less harmful for normal cells. Sulindac has been shown to be a potent chemo-protective agent against colorectal cancer in both human and animal models [25], while sulindac sulfide [18] and sulindac and its two metabolites [18], [28]] have been reported to upregulate the expression of carcinogen detoxification enzymes, including NQO1. It is known that sulindac compounds inhibit the activity of COX-1 and COX-2, and thus block the biosynthesis of prostaglandins [58]-[60]. In *in vivo* studies, sulindac has been shown to be reversibly reduced to sulindac sulfide, which can be irreversibly oxidized to sulindac sulfone, all three of which are anti-inflammatory. Since 1995, several clinical trials have established that sulindac is effective at reducing the number and size of polyps in patients with familial adenomatous polyposis, a precursor to colorectal cancer (NCI-P97-0110, NCI-P00-0150 [61]). Sulindac is a ligand of the aryl hydrocarbon receptor (AhR), an xenobiotic-sensing nuclear receptor that can be activated by chemical structures containing planar aromatic hydrocarbons, and thus evokes a cellular response that to detoxify xenobiotics. AhR activation leads to transcriptional upregulation of the NQO1 gene [62], [63]. In previous studies [30], [64], [65], sulindac and its two metabolites have been used to treat cancer cells at concentrations of 200 µM –1 mM, i.e. the concentrations used in our present study. In addition to reducing the growth of polyps, all three increase NQO1 activity and expression in colon cancer cells [28], and might therefore be good candidates to increase the cytotoxic effect of β-lapachone against lung cancer cells. When two cancer cell lines, CL1-1 and CL1-5, with low NQO1 expression and activity, were co-incubated with sulindac or its metabolites and β-lapachone, much higher cell death was seen with the CL1-5 cells than the CL1-1 cells (Figure 6 and 7). These results demonstrated that the effect of sulindac and its metabolites in upregulating NQO1 was greater in CL1-5 cells, which has lower NQO1 level and activity than CL1-1 cells, showing that sulindac and its metabolites can be used to increase the β-lapachone sensitivity of cells with lower NQO1 levels.

Many other compounds, such as toxifolin [32] and resveratrol [66], can increase NQO1 expression or activity, but are not FDA-approved. A search is underway for other compounds that can increase the activity or expression of NQO1 using high-throughput library screening, and two compounds, DMEBP and TRES, were recently found to be potent NQO1 inducers with low toxicity [3]. These compounds may also be valuable in increasing β-lapachone cytotoxicity for cancer cells with low NQO1 expression or activity.

### The NQO1 Inhibitor Dicoumarol or Transfection with NQO1 siRNA Inhibits the Effect of Sulindac on β-lapachone Toxicity for Lung Cancer Cells

Dicoumarol is widely used as a specific pharmacologic inhibitor of NQO1 and has been shown to inhibit both enzyme activity and expression [45], [67], [68]. NQO1 siRNA, designed to specifically target NQO1 mRNA, can lower the expression of NQO1 mRNA and protein. In our study, both agents blocked the synergistic effect of sulindac or its metabolites and β-lapachone on decreasing the survival of CL1-1 or CL1-5 cells.

Although β-lapachone is very toxic for many cancer cells, cells with lower NQO1 levels are less sensitive. However, from the present study, we can conclude that sulindac and its metabolites increase NQO1 expression and enzyme activity, and thus are potential synergistic drugs that might be used in combination with β-lapachone to treat cancer cells with high resistance to β-lapachone cytotoxicity.

## Supporting Information

Figure S1 **β-lapachone causes cell death of CL1-1 and CL1-5 cells by decreasing the mitochondrial membrane potential.** (A) Cells were left untreated or were incubated with 5 µM β-lapachone for the indicated time, and then the cell cycle distribution was analyzed using propidium iodide staining and flow cytometry. (B) Cells were incubated with 5 µM β-lapachone for the indicated time, then pro-caspase 3 and caspase 3 levels were analyzed by Western blotting. (C) Cells were incubated with 5 µM β-lapachone for the indicated time, then the mitochondrial membrane potential (MMP) was measured using the dye JC1 (Life Technology) and flow cytometry. (D) Cells were incubated with 5 µM β-lapachone for the indicated time, and then intracellular H_2_O_2_ levels were measured.
(TIF)

Figure S2 **zVAD-FMK, ALLM and ALLN do not block the cytotoxicity of β-lapachone.** CL1-1 cells (A) or CL1-5 cells (B) were left untreated or were incubated for 1 h with the indicated concentration of the pan caspase inhibitor zVAD (left panels) or the calpain inhibitor ALLM (center panels) or ALLN (right panels), then 5 µM β-lapachone was added for 12 or 24 h and cell viability measured using the MTT assay and expressed as percentage survival compared to the untreated cells.
(TIF)

Figure S3 **Dicoumarol, an NQO1 inhibitor, inhibits NQO1 activity and blocks the increase in intracellular calcium levels induced by β-lapachone.** (A) CL1-1 cells (left) or CL1-5 cells (right) were left untreated (CTL) or were incubated with 10 µM dicoumarol for 6 h, then NQO1 activity was measured. (B) CL1-1 cells (top panel) or CL1-5 cells (bottom panel) were left untreated or were incubated with 10 µM dicoumarol and/or 5 µM β-lapachone for 1 h, then were stained with Fluo-4 and the intensity of the Fluo-4 fluorescence measured by flow cytometry.
(TIF)

Figure S4 **Sulindac and its metabolites do not affect survival of lung cancer cells.** CL1-1, CL1-5, or A549 cells were left untreated or were incubated for 54 h with 100 or 250 µM sulindac (left panel) or sulindac sulfone (center panel) or for 12 h with 100 or 250 µM sulindac sulfide (right panel), then cell survival was measured by the MTT assay and expressed as percentage survival compared to the untreated cells.
(TIF)

Figure S5 **The cytotoxic effect of β-lapachone on A549 cells is enhanced by sulindac and its metabolites.** Two sets of cells were left untreated or were incubated for 6 h with the indicated concentration of sulindac, sulindac sulfone, or sulindac sulfide, then 2 µM β-lapachone was added to one set and incubation continued for 12 h, when cell survival was measured using crystal violet staining and expressed as percentage survival compared to the untreated cells.
(TIF)

Figure S6 **NQO1 siRNA has no effect on cell morphology or cell growth.** CL1-1 cells (top) and CL1-5 (bottom) were transfected with negative siRNA or NQO1 siRNA for 1 to 3 days, then pictures were taken using a digital camera and phase contrast microscopy. The scale bar represents 50 µm.
(TIF)

Figure S7 **NQO1 RNA levels are decreased by siRNA targeting NQO1.** A549, CL1-1, or CL1-5 cells were transfected for 48 h with siRNA targeting NQO1 (siNQO1) or control siRNA (siNeg), and then NQO1 mRNA levels were measured by realtime PCR and expressed as a fold change compared to the value for CL1-5 cells transfected with siNeg. * : p<0.05 compared to the result for the corresponding siNeg-transfected cells.
(TIF)

Table S1 **Primers used in the realtime PCR for actin and NQO1.**
(TIF)

Materials and Methods S1 (DOCX)

## References

[1] Pardee AB, Li YZ, Li CJ (2002) Cancer therapy with beta-lapachone. Curr Cancer Drug Targets 2: 227–242. 12188909 10.2174/1568009023333854

[2] Tagliarino C, Pink JJ, Reinicke KE, Simmers SM, Wuerzberger-Davis SM, et al (2003) Mu-calpain activation in beta-lapachone-mediated apoptosis. Cancer Biol Ther 2: 141–152. 12750552 10.4161/cbt.2.2.237

[3] Tan XL, Marquardt G, Massimi AB, Shi M, Han W, et al (2012) High-throughput library screening identifies two novel NQO1 inducers in human lung cells. Am J Respir Cell Mol Biol 46: 365–371. 22021338 10.1165/rcmb.2011-0301OC PMC3326428

[4] Minami T, Adachi M, Kawamura R, Zhang Y, Shinomura Y, et al (2005) Sulindac enhances the proteasome inhibitor bortezomib-mediated oxidative stress and anticancer activity. Clin Cancer Res 11: 5248–5256. 16033843 10.1158/1078-0432.CCR-05-0085

[5] Terai K, Dong GZ, Oh ET, Park MT, Gu Y, et al (2009) Cisplatin enhances the anticancer effect of beta-lapachone by upregulating NQO1. Anticancer Drugs 20: 901–909. 19738461 10.1097/CAD.0b013e328330098d

[6] Tagliarino C, Pink JJ, Dubyak GR, Nieminen AL, Boothman DA (2001) Calcium is a key signaling molecule in beta-lapachone-mediated cell death. J Biol Chem 276: 19150–19159. 11279125 10.1074/jbc.M100730200

[7] Bentle MS, Reinicke KE, Dong Y, Bey EA, Boothman DA (2007) Nonhomologous end joining is essential for cellular resistance to the novel antitumor agent, beta-lapachone. Cancer Res 67: 6936–6945. 17638905 10.1158/0008-5472.CAN-07-0935

[8] Planchon SM, Pink JJ, Tagliarino C, Bornmann WG, Varnes ME, et al (2001) beta-Lapachone-induced apoptosis in human prostate cancer cells: involvement of NQO1/xip3. Exp Cell Res 267: 95–106. 11412042 10.1006/excr.2001.5234

[9] Reinicke KE, Bey EA, Bentle MS, Pink JJ, Ingalls ST, et al (2005) Development of beta-lapachone prodrugs for therapy against human cancer cells with elevated NAD(P)H:quinone oxidoreductase 1 levels. Clin Cancer Res 11: 3055–3064. 15837761 10.1158/1078-0432.CCR-04-2185

[10] de Witte NV, Stoppani AO, Dubin M (2004) 2-Phenyl-beta-lapachone can affect mitochondrial function by redox cycling mediated oxidation. Arch Biochem Biophys 432: 129–135. 15542051 10.1016/j.abb.2004.09.020

[11] Pink JJ, Wuerzberger-Davis S, Tagliarino C, Planchon SM, Yang X, et al (2000) Activation of a cysteine protease in MCF-7 and T47D breast cancer cells during beta-lapachone-mediated apoptosis. Exp Cell Res 255: 144–155. 10694431 10.1006/excr.1999.4790

[12] Choi EK, Terai K, Ji IM, Kook YH, Park KH, et al (2007) Upregulation of NAD(P)H:quinone oxidoreductase by radiation potentiates the effect of bioreductive beta-lapachone on cancer cells. Neoplasia 9: 634–642. 17786182 10.1593/neo.07397 PMC1950433

[13] Lemasters JJ, Nieminen AL, Qian T, Trost LC, Elmore SP, et al (1998) The mitochondrial permeability transition in cell death: a common mechanism in necrosis, apoptosis and autophagy. Biochim Biophys Acta 1366: 177–196. 9714796 10.1016/s0005-2728(98)00112-1

[14] Smaili SS, Hsu YT, Youle RJ, Russell JT (2000) Mitochondria in Ca2+ signaling and apoptosis. J Bioenerg Biomembr 32: 35–46. 11768760 10.1023/a:1005508311495

[15] Belinsky M, Jaiswal AK (1993) NAD(P)H:quinone oxidoreductase1 (DT-diaphorase) expression in normal and tumor tissues. Cancer Metastasis Rev 12: 103–117. 8375015 10.1007/BF00689804

[16] Satsu H, Chidachi E, Hiura Y, Ogiwara H, Gondo Y, et al.. (2012) Induction of NAD(P)H:quinone oxidoreductase 1 expression by cysteine via Nrf2 activation in human intestinal epithelial LS180 cells. Amino Acids. 10.1007/s00726-012-1230-1 22302369

[17] Tsai CW, Lin CY, Wang YJ (2011) Carnosic acid induces the NAD(P)H: quinone oxidoreductase 1 expression in rat clone 9 cells through the p38/nuclear factor erythroid-2 related factor 2 pathway. J Nutr 141: 2119–2125. 22031657 10.3945/jn.111.146779

[18] Bottone FG Jr, Martinez JM, Collins JB, Afshari CA, Eling TE (2003) Gene modulation by the cyclooxygenase inhibitor, sulindac sulfide, in human colorectal carcinoma cells: possible link to apoptosis. J Biol Chem 278: 25790–25801. 12734198 10.1074/jbc.M301002200

[19] Dong GZ, Youn H, Park MT, Oh ET, Park KH, et al (2009) Heat shock increases expression of NAD(P)H:quinone oxidoreductase (NQO1), mediator of beta-lapachone cytotoxicity, by increasing NQO1 gene activity and via Hsp70-mediated stabilisation of NQO1 protein. Int J Hyperthermia 25: 477–487. 19657853 10.1080/02656730903049836

[20] Tugwell P, Ludwin D, Gent M, Roberts R, Bensen W, et al (1997) Interaction between cyclosporin A and nonsteroidal antiinflammatory drugs. J Rheumatol 24: 1122–1125. 9195520

[21] Muncie HL, Jr. (1986) Medical aspects of the multidisciplinary assessment and management of osteoarthritis. Clin Ther 9 Suppl B: 4–13. 3829096

[22] Fernandes E, Toste SA, Lima JL, Reis S (2003) The metabolism of sulindac enhances its scavenging activity against reactive oxygen and nitrogen species. Free Radic Biol Med 35: 1008–1017. 14572604 10.1016/s0891-5849(03)00437-4

[23] Karachalios GN, Donas G (1982) Sulindac in the treatment of acute gout arthritis. Int J Tissue React 4: 297–299. 7169301

[24] Klassen DK, Stout RL, Spilman PS, Whelton A (1989) Sulindac kinetics and effects on renal function and prostaglandin excretion in renal insufficiency. J Clin Pharmacol 29: 1037–1042. 2600190 10.1002/j.1552-4604.1989.tb03275.x

[25] Thun MJ, Henley SJ, Patrono C (2002) Nonsteroidal anti-inflammatory drugs as anticancer agents: mechanistic, pharmacologic, and clinical issues. J Natl Cancer Inst 94: 252–266. 11854387 10.1093/jnci/94.4.252

[26] Ruegg C, Zaric J, Stupp R (2003) Non steroidal anti-inflammatory drugs and COX-2 inhibitors as anti-cancer therapeutics: hypes, hopes and reality. Ann Med 35: 476–487. 14649330 10.1080/07853890310017053

[27] Hisamuddin IM, Wehbi MA, Chao A, Wyre HW, Hylind LM, et al (2004) Genetic polymorphisms of human flavin monooxygenase 3 in sulindac-mediated primary chemoprevention of familial adenomatous polyposis. Clin Cancer Res 10: 8357–8362. 15623613 10.1158/1078-0432.CCR-04-1073

[28] Ciolino HP, Bass SE, MacDonald CJ, Cheng RY, Yeh GC (2008) Sulindac and its metabolites induce carcinogen metabolizing enzymes in human colon cancer cells. Int J Cancer 122: 990–998. 17985343 10.1002/ijc.23218

[29] Resnick L, Rabinovitz H, Binninger D, Marchetti M, Weissbach H (2009) Topical sulindac combined with hydrogen peroxide in the treatment of actinic keratoses. J Drugs Dermatol 8: 29–32. 19180893

[30] Marchetti M, Resnick L, Gamliel E, Kesaraju S, Weissbach H, et al (2009) Sulindac enhances the killing of cancer cells exposed to oxidative stress. PLoS One 4: e5804. 19503837 10.1371/journal.pone.0005804 PMC2686156

[31] van Lieshout EM, Tiemessen DM, Peters WH, Jansen JB (1997) Effects of nonsteroidal anti-inflammatory drugs on glutathione S-transferases of the rat digestive tract. Carcinogenesis 18: 485–490. 9067546 10.1093/carcin/18.3.485

[32] Patten EJ, DeLong MJ (1999) Effects of sulindac, sulindac metabolites, and aspirin on the activity of detoxification enzymes in HT-29 human colon adenocarcinoma cells. Cancer Lett 147: 95–100. 10660094 10.1016/s0304-3835(99)00282-7

[33] Oberg M, Jaakkola MS, Woodward A, Peruga A, Pruss-Ustun A (2011) Worldwide burden of disease from exposure to second-hand smoke: a retrospective analysis of data from 192 countries. Lancet 377: 139–146. 21112082 10.1016/S0140-6736(10)61388-8

[34] Gan Q, Smith KR, Hammond SK, Hu TW (2007) Disease burden of adult lung cancer and ischaemic heart disease from passive tobacco smoking in China. Tob Control 16: 417–422. 18048620 10.1136/tc.2007.021477 PMC2807198

[35] Thill PG, Goswami P, Berchem G, Domon B (2011) Lung cancer statistics in Luxembourg from 1981 to 2008. Bull Soc Sci Med Grand Duche Luxemb: 43–55. 22272445

[36] Earle CC (2004) Outcomes research in lung cancer. J Natl Cancer Inst Monogr: 56–77. 10.1093/jncimonographs/lgh001 15504920

[37] Dienstmann R, Brana I, Rodon J, Tabernero J (2011) Toxicity as a biomarker of efficacy of molecular targeted therapies: focus on EGFR and VEGF inhibiting anticancer drugs. Oncologist 16: 1729–1740. 22135123 10.1634/theoncologist.2011-0163 PMC3248772

[38] Sunaga N, Shames DS, Girard L, Peyton M, Larsen JE, et al (2011) Knockdown of oncogenic KRAS in non-small cell lung cancers suppresses tumor growth and sensitizes tumor cells to targeted therapy. Mol Cancer Ther 10: 336–346. 21306997 10.1158/1535-7163.MCT-10-0750 PMC3061393

[39] Gupta AK, Soto DE, Feldman MD, Goldsmith JD, Mick R, et al (2004) Signaling pathways in NSCLC as a predictor of outcome and response to therapy. Lung 182: 151–162. 15526754 10.1007/s00408-004-0310-8

[40] Naime FF, Younes RN, Kersten BG, Anelli A, Beato CA, et al (2007) Metastatic non-small cell lung cancer in Brazil: treatment heterogeneity in routine clinical practice. Clinics (Sao Paulo) 62: 397–404. 17823701 10.1590/s1807-59322007000400005

[41] Spivack SD, Hurteau GJ, Fasco MJ, Kaminsky LS (2003) Phase I and II carcinogen metabolism gene expression in human lung tissue and tumors. Clin Cancer Res 9: 6002–6011. 14676126

[42] Kolesar JM, Pritchard SC, Kerr KM, Kim K, Nicolson MC, et al (2002) Evaluation of NQO1 gene expression and variant allele in human NSCLC tumors and matched normal lung tissue. Int J Oncol 21: 1119–1124. 12370763

[43] Chu YW, Yang PC, Yang SC, Shyu YC, Hendrix MJ, et al (1997) Selection of invasive and metastatic subpopulations from a human lung adenocarcinoma cell line. Am J Respir Cell Mol Biol 17: 353–360. 9308922 10.1165/ajrcmb.17.3.2837

[44] Pink JJ, Planchon SM, Tagliarino C, Varnes ME, Siegel D, et al (2000) NAD(P)H:Quinone oxidoreductase activity is the principal determinant of beta-lapachone cytotoxicity. J Biol Chem 275: 5416–5424. 10681517 10.1074/jbc.275.8.5416

[45] Lien YC, Kung HN, Lu KS, Jeng CJ, Chau YP (2008) Involvement of endoplasmic reticulum stress and activation of MAP kinases in beta-lapachone-induced human prostate cancer cell apoptosis. Histol Histopathol 23: 1299–1308. 18785111 10.14670/HH-23.1299

[46] Steelman LS, Chappell WH, Abrams SL, Kempf RC, Long J, et al (2011) Roles of the Raf/MEK/ERK and PI3K/PTEN/Akt/mTOR pathways in controlling growth and sensitivity to therapy-implications for cancer and aging. Aging (Albany NY) 3: 192–222. 21422497 10.18632/aging.100296 PMC3091517

[47] Kutuk O, Basaga H (2007) Apoptosis signalling by 4-hydroxynonenal: a role for JNK-c-Jun/AP-1 pathway. Redox Rep 12: 30–34. 17263905 10.1179/135100007X162329

[48] Saeki K, Kobayashi N, Inazawa Y, Zhang H, Nishitoh H, et al (2002) Oxidation-triggered c-Jun N-terminal kinase (JNK) and p38 mitogen-activated protein (MAP) kinase pathways for apoptosis in human leukaemic cells stimulated by epigallocatechin-3-gallate (EGCG): a distinct pathway from those of chemically induced and receptor-mediated apoptosis. Biochem J 368: 705–720. 12206715 10.1042/BJ20020101 PMC1223028

[49] Lee H, Park MT, Choi BH, Oh ET, Song MJ, et al (2011) Endoplasmic reticulum stress-induced JNK activation is a critical event leading to mitochondria-mediated cell death caused by beta-lapachone treatment. PLoS One 6: e21533. 21738692 10.1371/journal.pone.0021533 PMC3127577

[50] Orrenius S, Zhivotovsky B, Nicotera P (2003) Regulation of cell death: the calcium-apoptosis link. Nat Rev Mol Cell Biol 4: 552–565. 12838338 10.1038/nrm1150

[51] Cresteil T, Jaiswal AK (1991) High levels of expression of the NAD(P)H:quinone oxidoreductase (NQO1) gene in tumor cells compared to normal cells of the same origin. Biochem Pharmacol 42: 1021–1027. 1651729 10.1016/0006-2952(91)90284-c

[52] Schlager JJ, Powis G (1990) Cytosolic NAD(P)H:(quinone-acceptor)oxidoreductase in human normal and tumor tissue: effects of cigarette smoking and alcohol. Int J Cancer 45: 403–409. 2307529 10.1002/ijc.2910450304

[53] Smitskamp-Wilms E, Giaccone G, Pinedo HM, van der Laan BF, Peters GJ (1995) DT-diaphorase activity in normal and neoplastic human tissues; an indicator for sensitivity to bioreductive agents? Br J Cancer 72: 917–921. 7547240 10.1038/bjc.1995.433 PMC2034035

[54] Marin A, Lopez de Cerain A, Hamilton E, Lewis AD, Martinez-Penuela JM, et al (1997) DT-diaphorase and cytochrome B5 reductase in human lung and breast tumours. Br J Cancer 76: 923–929. 9328153 10.1038/bjc.1997.485 PMC2228079

[55] Berger MS, Talcott RE, Rosenblum ML, Silva M, AliOsman F, et al (1985) Use of quinones in brain-tumor therapy: preliminary results of preclinical laboratory investigations. J Toxicol Environ Health 16: 713–719. 2419579 10.1080/15287398509530781

[56] Park EJ, Choi KS, Kwon TK (2011) beta-Lapachone-induced reactive oxygen species (ROS) generation mediates autophagic cell death in glioma U87 MG cells. Chem Biol Interact 189: 37–44. 21035436 10.1016/j.cbi.2010.10.013

[57] Suzuki M, Amano M, Choi J, Park HJ, Williams BW, et al (2006) Synergistic effects of radiation and beta-lapachone in DU-145 human prostate cancer cells in vitro. Radiat Res 165: 525–531. 16669706 10.1667/RR3554.1

[58] Vane JR, Flower RJ, Botting RM (1990) History of aspirin and its mechanism of action. Stroke 21: IV12–23. 2124385

[59] Horton JK, Williams AS, Smith-Phillips Z, Martin RC, O’Beirne G (1999) Intracellular measurement of prostaglandin E2: effect of anti-inflammatory drugs on cyclooxygenase activity and prostanoid expression. Anal Biochem 271: 18–28. 10361000 10.1006/abio.1999.4118

[60] Waddell WR, Loughry RW (1983) Sulindac for polyposis of the colon. J Surg Oncol 24: 83–87. 6887943 10.1002/jso.2930240119

[61] PDQ Clinical Trail Database, National Cancer Institure.

[62] Ciolino HP, MacDonald CJ, Memon OS, Bass SE, Yeh GC (2006) Sulindac regulates the aryl hydrocarbon receptor-mediated expression of Phase 1 metabolic enzymes in vivo and in vitro. Carcinogenesis 27: 1586–1592. 16531450 10.1093/carcin/bgi359

[63] Lin X, Yang H, Zhou L, Guo Z (2011) Nrf2-dependent induction of NQO1 in mouse aortic endothelial cells overexpressing catalase. Free Radic Biol Med 51: 97–106. 21569840 10.1016/j.freeradbiomed.2011.04.020 PMC3109219

[64] Yamamoto Y, Yin MJ, Lin KM, Gaynor RB (1999) Sulindac inhibits activation of the NF-kappaB pathway. J Biol Chem 274: 27307–27314. 10480951 10.1074/jbc.274.38.27307

[65] Zhang T, Fields JZ, Ehrlich SM, Boman BM (2004) The chemopreventive agent sulindac attenuates expression of the antiapoptotic protein survivin in colorectal carcinoma cells. J Pharmacol Exp Ther 308: 434–437. 14610217 10.1124/jpet.103.059378

[66] Hebbar V, Shen G, Hu R, Kim BR, Chen C, et al (2005) Toxicogenomics of resveratrol in rat liver. Life Sci 76: 2299–2314. 15748624 10.1016/j.lfs.2004.10.039

[67] Gustafson DL, Beall HD, Bolton EM, Ross D, Waldren CA (1996) Expression of human NAD(P)H: quinone oxidoreductase (DT-diaphorase) in Chinese hamster ovary cells: effect on the toxicity of antitumor quinones. Mol Pharmacol 50: 728–735. 8863816 PMC3883621

[68] Cross JV, Deak JC, Rich EA, Qian Y, Lewis M, et al (1999) Quinone reductase inhibitors block SAPK/JNK and NFkappaB pathways and potentiate apoptosis. J Biol Chem 274: 31150–31154. 10531305 10.1074/jbc.274.44.31150

