# Supplementary figures and images for: Sulindac Compounds Facilitate the Cytotoxicity of β-Lapachone by Up-Regulation of NAD(P)H Quinone Oxidoreductase in Human Lung Cancer Cells
Source: PLoS One. 2014 Feb 5;9(2):e88122. doi: 10.1371/journal.pone.0088122 (PMC3914905; doi:10.1371/journal.pone.0088122)

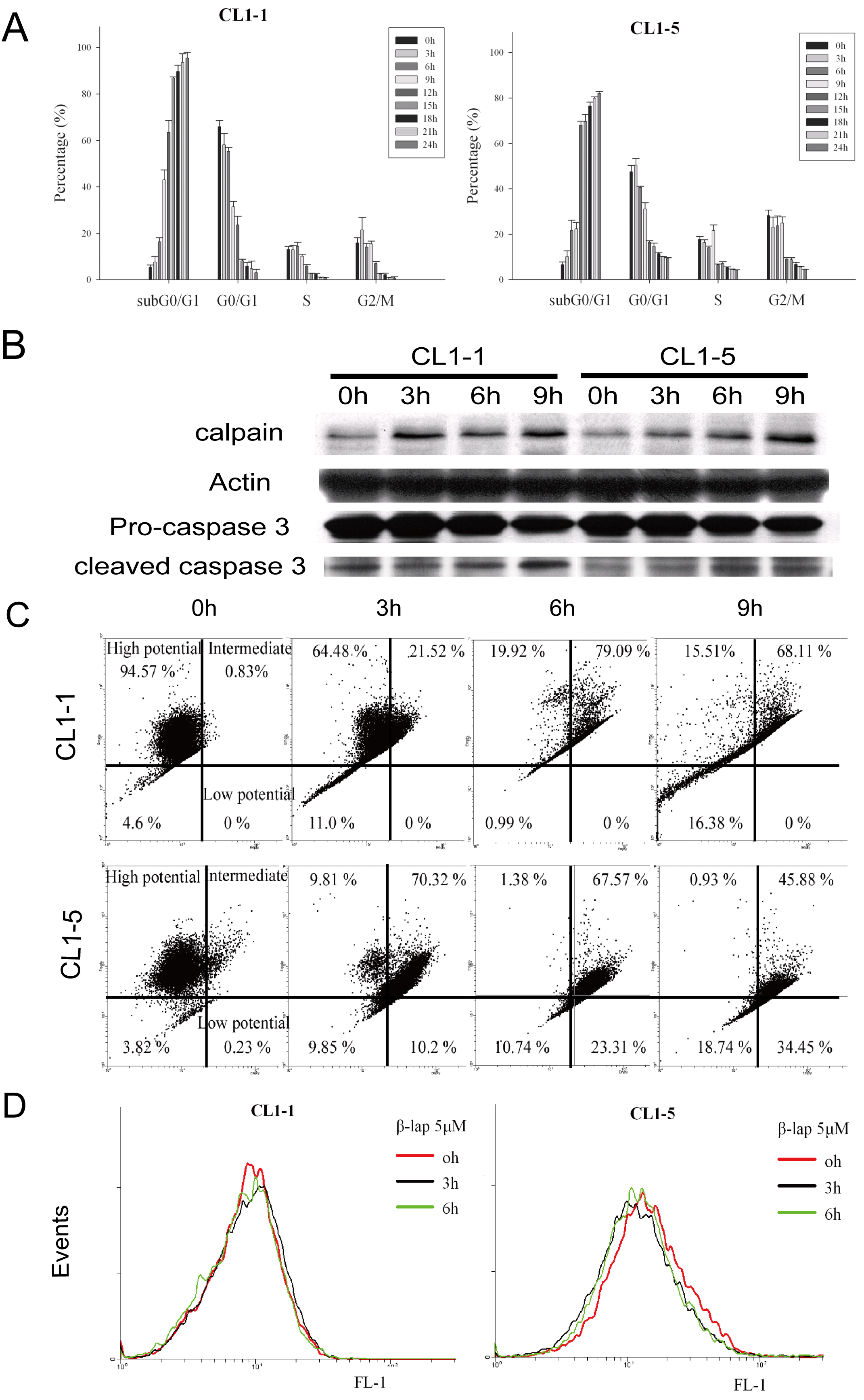

Supplement: Figure S1 — β-lapachone causes cell death of CL1-1 and CL1-5 cells by decreasing the mitochondrial membrane potential. (A) Cells were left untreated or were incubated with 5 µM β-lapachone for the indicated time, and then the cell cycle distribution was analyzed using propidium iodide staining and flow cytometry. (B) Cells were incubated with 5 µM β-lapachone for the indicated time, then pro-caspase 3 and caspase 3 levels were analyzed by Western blotting. (C) Cells were incubated with 5 µM β-lapachone for the indicated time, then the mitochondrial membrane potential (MMP) was measured using the dye JC1 (Life Technology) and flow cytometry. (D) Cells were incubated with 5 µM β-lapachone for the indicated time, and then intracellular H2O2 levels were measured. (TIF) [file pone.0088122.s001.tif]

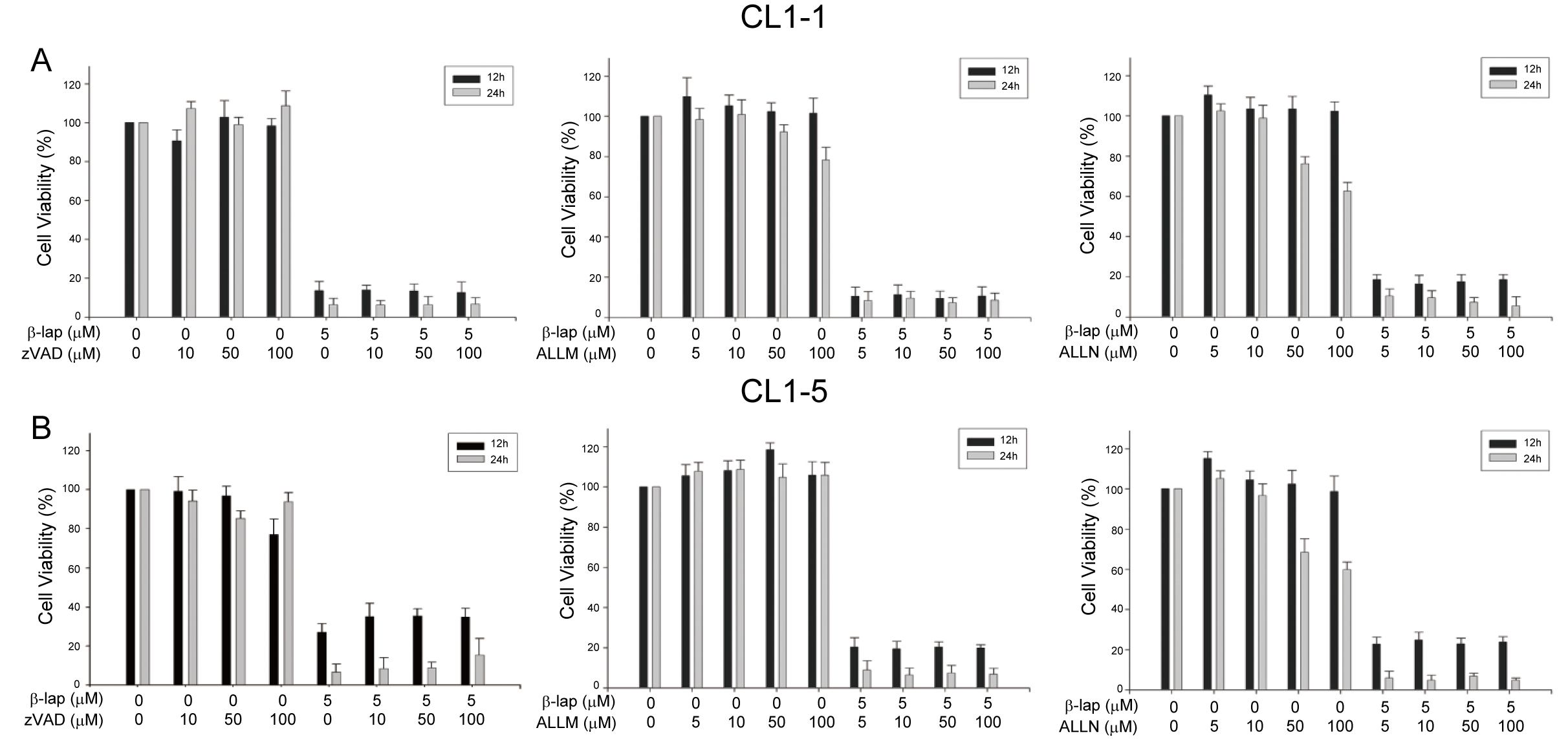

Supplement: Figure S2 — zVAD-FMK, ALLM and ALLN do not block the cytotoxicity of β-lapachone. CL1-1 cells (A) or CL1-5 cells (B) were left untreated or were incubated for 1 h with the indicated concentration of the pan caspase inhibitor zVAD (left panels) or the calpain inhibitor ALLM (center panels) or ALLN (right panels), then 5 µM β-lapachone was added for 12 or 24 h and cell viability measured using the MTT assay and expressed as percentage survival compared to the untreated cells. (TIF) [file pone.0088122.s002.tif]

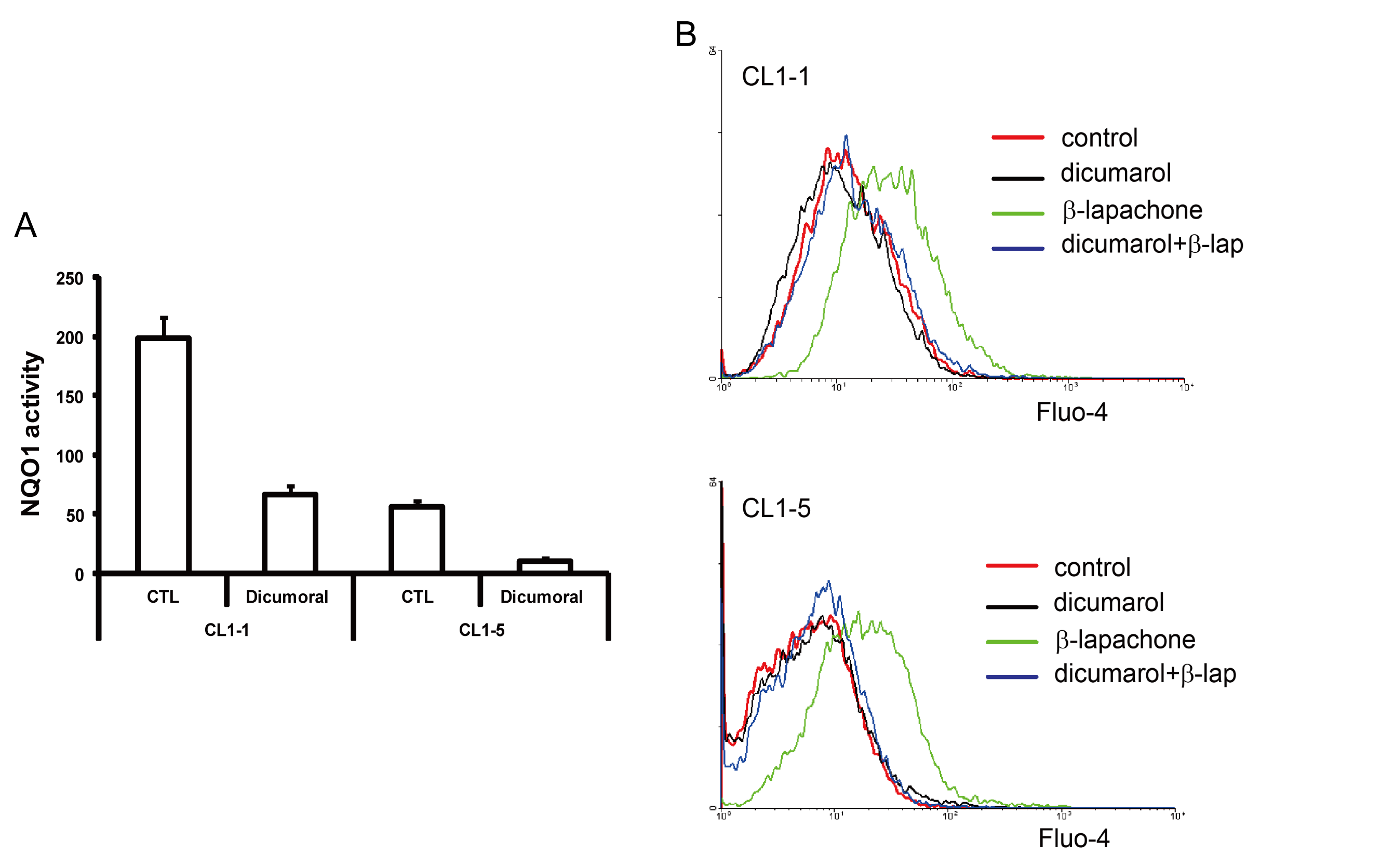

Supplement: Figure S3 — Dicoumarol, an NQO1 inhibitor, inhibits NQO1 activity and blocks the increase in intracellular calcium levels induced by β-lapachone. (A) CL1-1 cells (left) or CL1-5 cells (right) were left untreated (CTL) or were incubated with 10 µM dicoumarol for 6 h, then NQO1 activity was measured. (B) CL1-1 cells (top panel) or CL1-5 cells (bottom panel) were left untreated or were incubated with 10 µM dicoumarol and/or 5 µM β-lapachone for 1 h, then were stained with Fluo-4 and the intensity of the Fluo-4 fluorescence measured by flow cytometry. (TIF) [file pone.0088122.s003.tif]

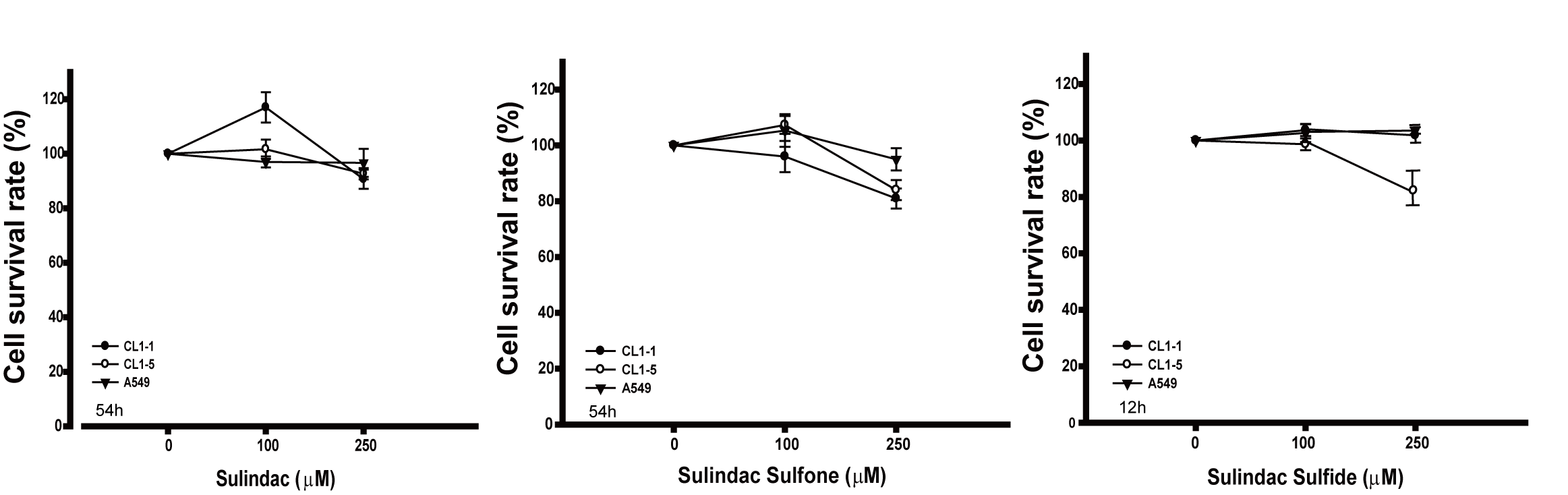

Supplement: Figure S4 — Sulindac and its metabolites do not affect survival of lung cancer cells. CL1-1, CL1-5, or A549 cells were left untreated or were incubated for 54 h with 100 or 250 µM sulindac (left panel) or sulindac sulfone (center panel) or for 12 h with 100 or 250 µM sulindac sulfide (right panel), then cell survival was measured by the MTT assay and expressed as percentage survival compared to the untreated cells. (TIF) [file pone.0088122.s004.tif]

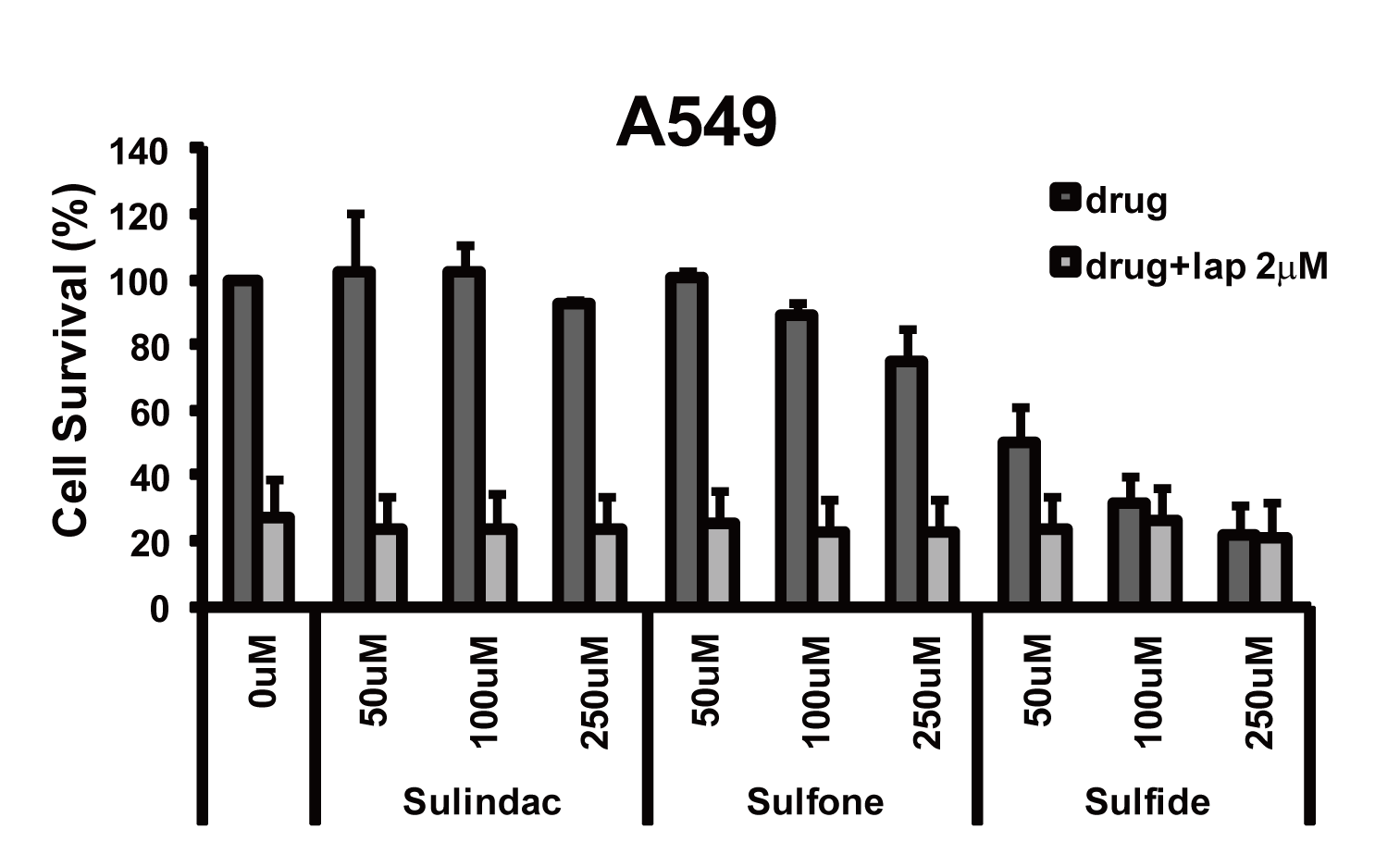

Supplement: Figure S5 — The cytotoxic effect of β-lapachone on A549 cells is enhanced by sulindac and its metabolites. Two sets of cells were left untreated or were incubated for 6 h with the indicated concentration of sulindac, sulindac sulfone, or sulindac sulfide, then 2 µM β-lapachone was added to one set and incubation continued for 12 h, when cell survival was measured using crystal violet staining and expressed as percentage survival compared to the untreated cells. (TIF) [file pone.0088122.s005.tif]

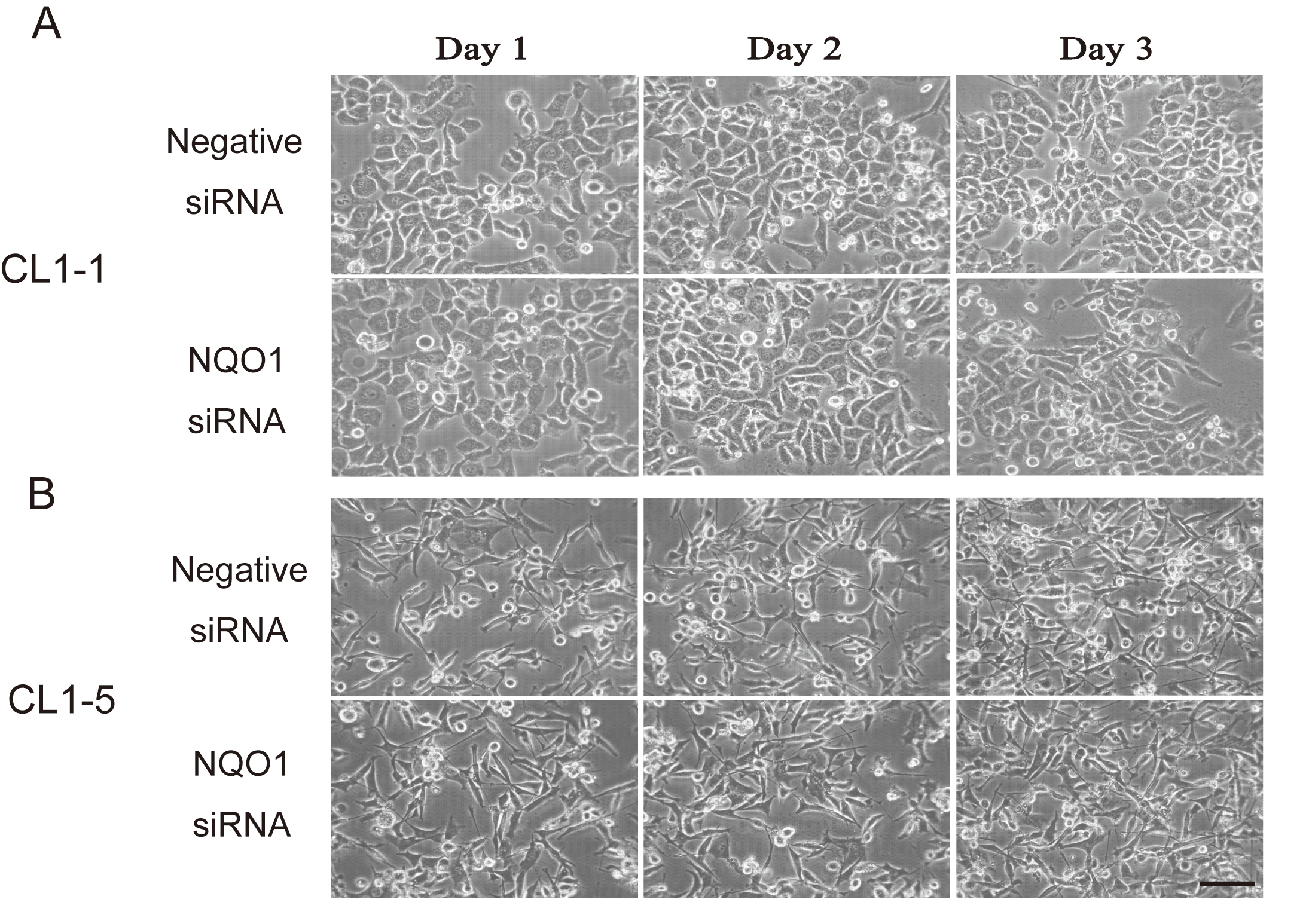

Supplement: Figure S6 — NQO1 siRNA has no effect on cell morphology or cell growth. CL1-1 cells (top) and CL1-5 (bottom) were transfected with negative siRNA or NQO1 siRNA for 1 to 3 days, then pictures were taken using a digital camera and phase contrast microscopy. The scale bar represents 50 µm. (TIF) [file pone.0088122.s006.tif]

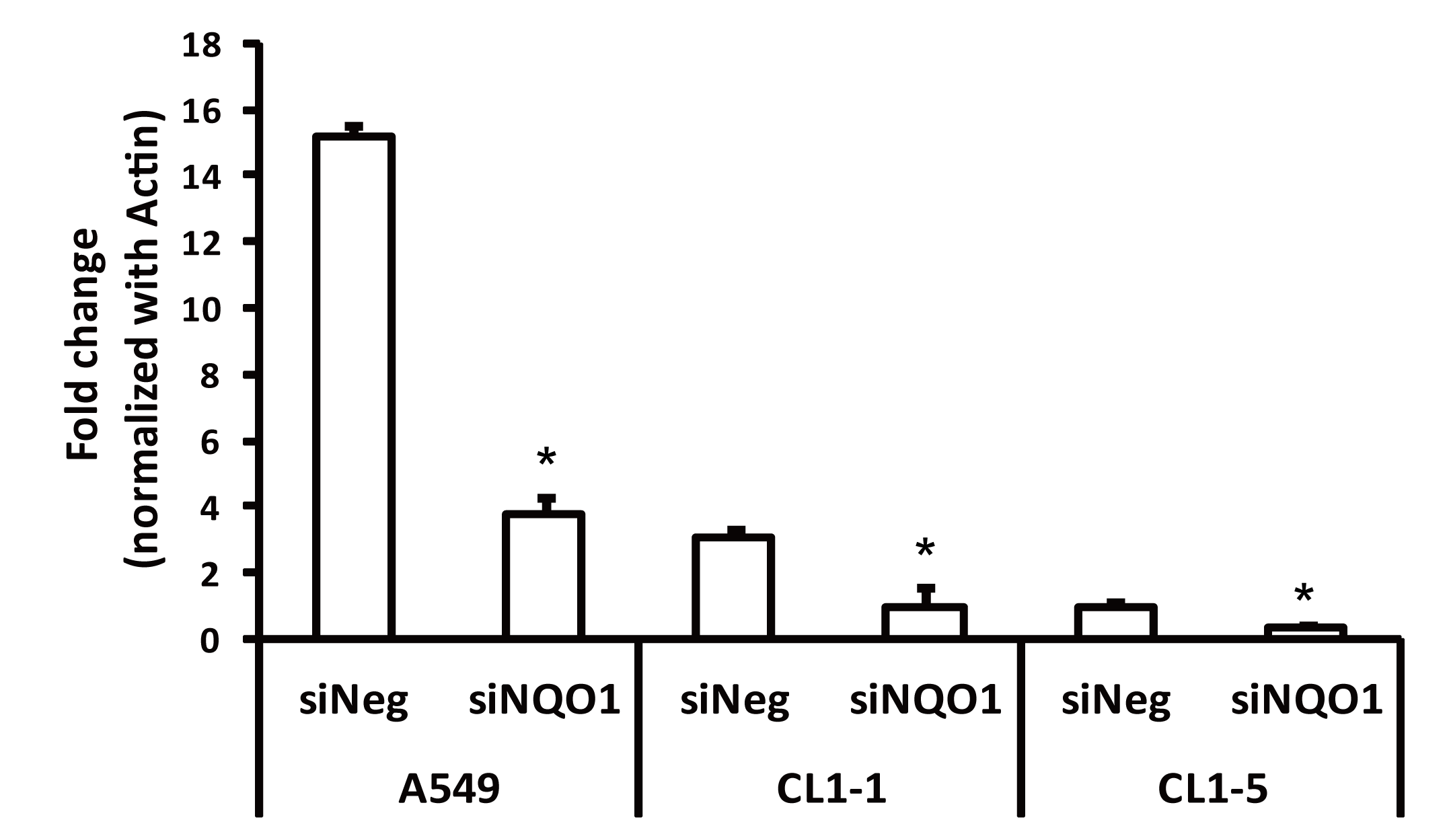

Supplement: Figure S7 — NQO1 RNA levels are decreased by siRNA targeting NQO1. A549, CL1-1, or CL1-5 cells were transfected for 48 h with siRNA targeting NQO1 (siNQO1) or control siRNA (siNeg), and then NQO1 mRNA levels were measured by realtime PCR and expressed as a fold change compared to the value for CL1-5 cells transfected with siNeg. * : p<0.05 compared to the result for the corresponding siNeg-transfected cells. (TIF) [file pone.0088122.s007.tif]

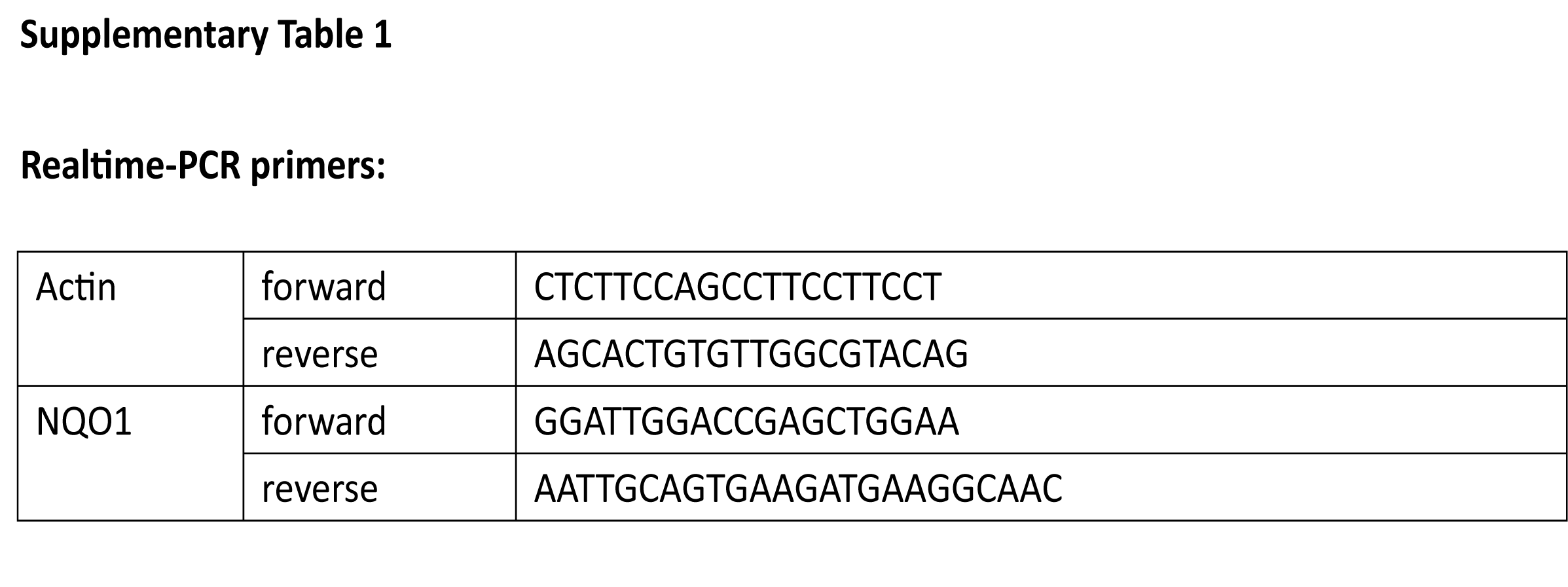

Supplement: Table S1 — Primers used in the realtime PCR for actin and NQO1. (TIF) [file pone.0088122.s008.tif]
