# Supplementary material for: Sulindac Compounds Facilitate the Cytotoxicity of β-Lapachone by Up-Regulation of NAD(P)H Quinone Oxidoreductase in Human Lung Cancer Cells
Source: PLoS One. 2014 Feb 5;9(2):e88122. doi: 10.1371/journal.pone.0088122 (PMC3914905; doi:10.1371/journal.pone.0088122)
Supplement: Materials and Methods S1 — (DOCX) [file pone.0088122.s009.docx]

**Supporting Information Materials and Methods**

**Intracellular H_2_O_2_ detection**

Cells were treated with drugs for indicated time and incubated with DCFHDA (5 μM) (Molecular Probes) for 30 min at 37℃. Cells were than detached by trypsin and analyzed by flow cytometry with FL1 parameter.

**Cell Cycle analysis**

Cells were treated with 5 μM β-lapachone for indicated times, collected, and store at -20 ℃ in 80 % EtOH/PBS for 2 hours. Cells were then washed twice, incubated for 30 min at 37 ℃ with 0.5 ml of 0.5 % Triton X-100/PBS containing 1 mg/ml of RNase A, and stained for 10 min with 0.5 ml of 50 μg/ml propidium iodide (PI). The intensity of the fluorescence emitted by the PI-DNA complex was quantified after laser excitation of the fluorescent dye, using a FACScan flow cytometry.

**Mitochondrial membrane potential measurement**

Cells were seeded into a 6-well culture plate for 24 h, and then treated with 5 μM β-lapachone.Cells were then incubated in medium containing 10 mg/ml of 5,50,6,60-tetrachloro-1,10,3,30-tetraethyl-benzimidazolylcarbocyanine iodide (JC-1) at 37 ℃ for 30 min, washed with PBS, trypsinized, and immediately analyzed by

flow cytometry.

**Real-time RT-PCR**

Total RNA was reverse-transcribed to cDNA with SuperScript II reverse transcription kit, then used for real-time PCR with Power SYBR Green PCR Mix (Applied Biosystems) and indicated primers (Supplementary [Table 1](http://www.plosgenetics.org/article/info%3Adoi%2F10.1371%2Fjournal.pgen.1002229#pgen.1002229.s009)), and normalized to β-actin mRNA levels measured in parallel.
